# Butenolide derivatives from *Aspergillus terreus* selectively inhibit butyrylcholinesterase

**DOI:** 10.3389/fchem.2022.1063284

**Published:** 2022-12-21

**Authors:** Xiang Cui, Shanshan Deng, Guoyin Li, Yunxia Zhang, Lining Wang, Changjing Wu, Yanru Deng

**Affiliations:** ^1^ College of Traditional Chinese Medicine, Tianjin University of Traditional Chinese Medicine, Tianjin, China; ^2^ College of Life Sciences and Agronomy, Zhoukou Normal University, Zhoukou, China

**Keywords:** Aspergillus terreus, secondary metabolites, butenolide derivatives, structure elucidation, butyrylcholinesterase inhibitor

## Abstract

Two undescribed butenolide derivatives, asperteretal J (**1**) and K (**2**), together with 13 known ones (**3**–**15**) were isolated from an endophytic fungus *Aspergillus terreus* SGP-1, the fermentation product of which exhibited selective inhibitory activity toward butyrylcholinesterase. The structures of the new compounds were elucidated based on HRMS and NMR data, and the absolute configurations were determined by specific optical rotation comparison. All compounds were evaluated for cholinesterase inhibitory effects with galantamine as a positive control. Compounds **4**–**8** selectively inhibited butyrylcholinesterase with IC_50_ values of 18.4–45.8 µM in a competitive manner, with Ki values of 12.3–38.2 µM. The structure-activity relationship was discussed. Molecular docking and dynamic simulation of the inhibitor-enzyme complex were performed to better understand the interactions.

## Introduction

As the most common and ultimately fatal degenerative brain disorder, Alzheimer’s disease (AD) is characterized by central cognitive and behavioral deficits and has attracted extensive worldwide attention for its serious public health impact (Alzheimer’s Association Report, 2021). The World Alzheimer Report 2018 reported that 50 million people are affected by dementia, and that number will be tripled to 152 million by 2050 (Lane et al., 2018). Due to the complexity of AD pathology, the exact etiology of AD remains incompletely understood. There are diverse hypotheses explaining the pathogenesis and progression of AD, including cholinergic dysfunction (Craig et al., 2011), amyloid-β (Aβ) deposits (Goedert and Spillantini, 2006), tau protein aggregation (Grundke-Iqbal et al., 1986), neuroinflammation (Linker et al., 2011), and mitochondrial dysfunction (Aliev et al., 2014). However, only six drugs have been applied clinically for AD treatment, including four cholinesterase inhibitors (Marucci et al., 2021), one N-methyl-D-aspartate (NMDA) receptor antagonist, and one sodium oligomannate that remodels the gut microbiota and suppresses neuroinflammation (Wang et al., 2019). To date, inhibitors of cholinesterases (ChEs) remain as reasonable and promising therapeutic drugs for AD treatment.

The cholinergic hypothesis suggests that deficiency of acetylcholine (ACh) has a strong relationship with cognitive function, which is regarded as a pathological feature of AD (Bartus., 2000; Sun et al., 2022). There are two ChEs in the body, acetylcholinesterase (AChE, EC 3.1.1.7) and butyrylcholinesterase (BChE, EC 3.1.1.8). During the progression of AD, AChE levels in the brain were substantially reduced to 10%, while BChE increased to 165% of normal levels, functionalizing the compensation capacity of the latter for acetylcholine hydrolysis (Perry et al., 1978; Ballard et al., 2005). Additionally, dysfunction of BChE was found to produce few negative effects on holistic health (Manoharan et al., 2007; Li et al., 2008), and avoided the typical cholinergic toxicity in peripheral tissues induced by AChE inhibitors (Li et al., 2018). Therefore, BChE has been regarded as a potential therapeutic target for AD with safety advantages.

In recent years, many efforts have been devoted to discovering potent and selective BChE inhibitors through chemical design and synthesis (Li et al., 2021; Rossi et al., 2021), or screening of natural products (NPs) and their derivatives (Orhan et al., 2017; Jabir et al., 2018; Wu et al., 2019; Chen et al., 2021; Onder et al., 2022). Galantamine, isolated from the bulbs and the flowers of *Galanthus woronowii*, is an approved drug used for treating AD as a natural AChE inhibitor with relatively weak BChE inhibitory activity (Marucci et al., 2021). Fungal secondary metabolites are NPs of abundant chemical and bioactive diversity; however only a few studies screening for BChE inhibitors from fungal NPs were reported so far (Piemontese et al., 2018; Li et al., 2020; Lin et al., 2021). In the present study, we screened an *Aspergillus terreus* strain from eight endophytic fungal strains derived from the root bark of *Morus alba* L., the fermentation product of which exhibited potent selective BChE inhibitory activity. Further chemical investigation led to the isolation of 15 butanolide derivatives, including two new ones. ChE inhibition assays of the isolated compounds showed that some of them selectively inhibited BChE. Herein, we report the screening, isolation, structure determination, enzyme inhibition evaluation, and molecular docking and dynamic simulation of these natural products.

## Material and methods

### General experimental procedures

Sephadex™ LH-20 (GE Healthcare, Uppsala, Sweden), silica gel (200–300 mesh, Qingdao Marine Chemical Inc., Qingdao, China), and YMC*GEL^®^ ODS-A-HG (12 nm, S-50 μm, YMC Co., Ltd., Kyoto, Japan) were used for column chromatography (CC). The preparative high-performance liquid chromatography (HPLC) was performed on a QuikSep chromatographic system (H&E, Beijing, China), and a Gemini C18 column (21.2 mm × 250 mm, column temperature: 26°C) was used for separation and purification. Optical rotations were measured on a P-2000 digital polarimeter (JASCO, Tokyo, Japan). UV spectra were recorded on a UV-2600 spectrophotometer (Shimadzu, Kyoto, Japan). High-resolution electrospray ionization mass spectrometry (HR-ESI-MS) was measured on a Xevo G2-XS quadrupole time-of-flight (QTOF) mass spectrometer (Agilent, CA, United States), and all 1D and 2D NMR spectra were obtained on a Bruker-500 (500 MHz 1H and 125 MHz 13C-NMR) NMR spectrometer. Absorbance was read on a SynergyHTX micro plate reader (BioTek, VT, United States).

### Fungal material

Strain *Aspergillus terreus* SGP-1 together with seven other strains were isolated from the root bark of *Morus alba* L. collected from Zhoukou City, Henan Province, China in March 2022. The isolates were identified based on morphology and sequence analysis of the ITS regions of the rDNA (GenBank No. SUB12083846 for strain SGP-1). The fungal strains have been preserved in the culture collection center of the College of Life Sciences and Agronomy, Zhoukou Normal University.

### Screening of the bioactive fungal strain and constituents

The fresh spore suspensions of SGP-1 and the seven other strains were inoculated into an Erlenmeyer flask (250 ml) containing 100 ml of liquid medium (glucose 2%, maltose 1%, mannitol 2%, glutamic acid 1%, peptone 0.5%, and yeast extract 0.3% in distilled water) and fermented at 28°C for 12 days on a rotary shaker at 200 rpm. To each fermentation broth was added 200 ml of ethyl acetate (EtOAc), and extraction was performed with the assistance of ultrasonication (40 kHz) for 30 min. The EtOAc solutions were removed *in vacuo* at 37°C to produce the EtOAc extracts. Each fungal EtOAc extract was dissolved in methanol at a concentration of 100.0 mg/ml, and was further diluted to a 10.0 mg/ml solution used for an *in vitro* BChE inhibitory activity assay. A sample of strain SGP-1 was analyzed using an HPLC-photo-diode array detector (PDAD)-UV with an analytical Kromasil C18 column (5 μm, 100 Å, 4.6 mm × 250 mm; Akzo Nobel) on an Agilent 1100 HPLC system equipped with a PDAD (G1316A). The extraction solution in methanol (100.0 mg/ml) was filtered with a 0.45-μm membrane, and then injected (20 μl) into the column, followed by elution with an MeOH-H_2_O linear gradient (20%→100% MeOH in 20 min followed by 5 min with isocratic 100% MeOH) mobile phase (flow rate 1 ml/min). During the separation procedure, the constituents of the emerging chromatographic peaks were collected in Eppendorf tubes with 2 s of delay. Each eluate was fixed to a volume of 200 μl, which was then used for the BChE inhibitory activity assay. The acquired PDAD data, including the UV spectra of peaks, were processed using Agilent OpenLAB software.

### Chemical investigation of *A. terreus* SGP-1

The strain SGP-1 spore suspension was inoculated into five 1,000-ml Erlenmeyer flasks, each containing 400 ml of sterile liquid medium. The culture was grown at 28°C with rotating oscillations of 200 rpm for 48 h to produce the seed culture (2 L). The seed culture was further added into a fermentation cylinder containing the same sterile liquid medium (70 L), and was fermented at 28°C for 12 days, with the internal air pressure kept at 0.15 MP by an air compressor. The entire broth volume (68 L) was separated into the filtrate and the mycelial cake by filtration with gauze. The filtrate (62 L) was then subjected to an AB-8 macroporous resin column with a column volume (CV) of 2.4 L, and eluted using water and 95% ethanol successively. The 95% ethanol eluate (3 CVs) was collected for further processing. The mycelial cake was extracted twice with 95% ethanol (6 L) assisted by ultrasonication for 2 h, followed by filtration to produce the ethanol extract. All the ethanol solutions were combined and concentrated into a water suspension (approximately 3 L), which was further extracted with EtOAc to generate a total of 66 g of EtOAc extract. The separation of the butenolide derivatives was conducted under the guidance of the characteristic UV absorption by HPLC-PDAD-UV analysis.

The EtOAc extract (66 g) of strain SGP-1 was separated using silica gel CC, and a stepwise elution was conducted with b.p. 60°C–90°C petroleum ether (P), dichloro-methane (D), and methanol (M) to obtain nine fractions (Fr). HPLC analysis suggested that Fr-6 (31 g, eluted using D-M 95:5), Fr-8 (1.4 g, eluted using D-M 85:15), and Fr-9 (1.9 g, eluted using D-M 80:20) contained the butenolide derivatives. Fr-6 was subjected to Sephadex LH-20 CC and eluted using M to give eight subfractions, including Fr-6-7 (7.3 g), which was further separated using HPLC (methanol-H_2_O 70:30, 10 ml/min) to produce **14** (209 mg, *t*_R_ = 13.5 min) and **6** (2.3 g, *t*_R_ = 18.6 min). Fr-8 (1.4 g) was loaded on an ODS RP-C_18_ column with a gradient elution of aqueous methanol (20, 40, 60, 80, and 100%), and the subfraction Fr-8-2 (eluted using 60%–80% methanol) was separated using HPLC (methanol-H_2_O 62:38, 10 ml/min) to produce **2** (7.5 mg, *t*_R_ = 8.9 min), **12** (46 mg, *t*_R_ = 10.8 min), **1** (17.5 mg, *t*_R_ = 12.5 min), **13** (52 mg, *t*_R_ = 15.0 min), **4** (35 mg, *t*_R_ = 16.5 min), **8** (18 mg, *t*_R_ = 17.8 min), **3** (20 mg, *t*_R_ = 21.0 min), **7** (32 mg, *t*_R_ = 26.5 min), **10** (11.5 mg, *t*_R_ = 27.9 min), and **11** (74 mg, *t*_R_ = 31.5 min). Fr-9 (1.9 g) was also separated using ODS CC with the same gradient elution of aqueous methanol, and Fr-9-6 (0.7 g, eluted using 70% methanol) was purified using HPLC (methanol-H_2_O 65:36, 10 ml/min) to yield **9** (15 mg, *t*_R_ = 10.0 min) and **15** (13 mg, *t*_R_ = 18.9 min), while Fr-9-8 (0.3 g, eluted using 90% methanol) produced **5** (6.0 mg, *t*_R_ = 26.2 min) by semi-preparative HPLC (methanol-H_2_O 70:30, 10 ml/min).

Asperteretal J (**1**): pale yellow solid (MeOH), [α] ‒18.4 (c 0.15, MeOH). UV (MeOH) λmax (log ε): 203 (4.36), 226 (4.11), 309 (3.70) nm. Positive HR-ESI-MS: *m/z* measured 479.1678 [M + Na]^+^, calcd for C_25_H_28_O_8_Na [M + Na]^+^ 479.1682; negative HR-ESI-MS: *m/z* measured 455.1721 [M–H]^−^, calcd for C_25_H_27_O_8_ [M–H]^-^ 455.1706. For ^1^H NMR and ^13^C NMR spectroscopic data, see Table 1.

**TABLE 1:** 500 MHz ^1^H and 125 MHz^13^C NMR data of compounds **1** and **2** in CD_3_OD^a^.

No.	1		2	
	*δ*_C_	*δ*_H_ (J in Hz)	*δ*_C_	*δ*_H_ (J in Hz)
1	174.2 s	—	174.2 s	—
2	127.3 s	—	127.35 s	—
3	157.0 s	—	157.1 s	—
4	103.1 s	—	103.1 s	—
5	169.3 s	—	169.3 s	—
6	30.1 t	3.77 brs	30.1 t	Ha 3.81, d (15.6)
				Hb 3.73, d (15.6)
1′	122.1 s	—	122.1 s	—
2′	131.8 d	7.48, d (8.9)	131.8 d	7.47, d (8.9)
3′	116.6 d	6.78, d (8.9)	116.6 d	6.78, d (8.9)
4′	161.0 s	—	161.1 d	—
5′	116.6 d	6.78, d (8.9)	116.6 d	6.78, d (8.9)
6′	131.8 d	7.48, d (8.9)	131.8 d	7.47, d (8.9)
1″	129.1 s	—	129.2 s	—
2″	130.6 d	6.90, d (2.3)	130.5 d	6.92, d (2.3)
3″	130.5 s	—	130.7 s	—
4″	154.9 s	—	154.9 s	—
5″	116.1 d	6.67, d (8.2)	116.1 d	6.67, d (8.2)
6″	127.4 d	6.85, dd (8.2, 2.3)	127.32 d	6.85, dd (8.2, 2.3)
1‴	25.8 t	2.57–2.50, m	26.3 t	2.66–2.56, m
2‴	40.4 t	1.72–1.65, m	44.9 t	1.72–1.62, m
3‴	76.4 s	—	71.6 s	—
4‴	25.5 q	1.19, s	29.2 q	1.23, s
5‴	25.5 q	1.19, s	29.1 q	1.23, s
5-OCH_3_	54.1 q	3.74, s	54.1 q	3.75, s
3‴-OCH_3_	49.4 q	3.21, s	—	—

^a^ Chemical shift values were recorded using the solvent signal (CD_3_OD: *δ*_H_ 3.31, *δ*_C_ 49.00) as references.

Asperteretal K (**2**): pale yellow solid (MeOH), [α] ‒17.5 (c 0.22, MeOH). UV (MeOH) λmax (log ε): 202 (4.25), 228 (3.98), and 308 (3.68) nm. Positive HR-ESI-MS: *m/z* measured 465.1526 [M + Na]^+^, calcd for C_24_H_26_O_8_Na [M + Na]^+^ 465.1525; negative HR-ESI-MS: *m/z* measured 441.1548 [M–H]^−^, calcd for C_24_H_25_O_8_ [M–H]^−^ 441.1549. For ^1^H NMR and ^13^C NMR spectroscopic data, see Table 1.

### *In vitro* ChE inhibitory activity assay

The inhibitory activities against AChE (EC 3.1.1.7) and BChE (EC 3.1.1.8) of the fungal samples and test compounds were analyzed following Ellman’s method (Ellman et al., 1961) with modifications. AChE (EC 3.1.1.7, from electric eel), BChE (EC 3.1.1.8, from equine serum), acetylthiocholine iodide (ATCI), butyrylthiocholine iodide (BTCI), and 5,5′-dithiobis (2-nitrobenzoic acid) (DTNB) were purchased from Aladdin (Shanghai, China). AChE and BChE solutions were prepared with water (0.2 units/ml). ATCI and BTCI solutions (10 mM) were prepared with deionized water before using. A DTNB solution (10 mM) was prepared in a 0.1 M, pH 8.0 solution of potassium dihydrogen phosphate (1.36 g, 10 mmol) in 100 ml of water (PBS). The fungal samples for screening were diluted to 1.0 mg/ml with methanol. The test compounds were dissolved in methanol (10 mM) as stock solutions. Subsequently, at least five different concentrations of each compound (double ratio dilution with methanol) were used to determine the half maximal inhibitory concentration (IC_50_). The tests were performed using 96-well plates. First, 160 μl of PBS, 2 μl of the test sample solution, 20 μl of AChE or BChE, and 10 μl of DTNB were added and mixed in each well, and then preincubated at 37°C for 10 min. After the addition of 10 μl of ATCI or BTCI, the reaction was initiated and incubated at 37°C for another 10 min. Then, the absorbance was measured at 412 nm. The wells with 2 μl of methanol replacing the test sample solution was used to determine 100% of the enzyme activity, and 20 μl of water replacing the enzyme solution was used to determine the blank value. All of the tests for each sample were performed in triplicate. The inhibition curve was fitted by plotting the inhibition rate vs*.* the logarithm of the test compound concentration. The IC_50_ values were calculated using Microsoft Excel software, and the data were expressed as the mean ± SD. The inhibition rate was calculated using the formula: IR% = [(A_c_-A_s_)]/[(A_c_-A_b_)] × 100%, where A_b_ denotes the absorbance of the blank control without the enzyme solution, A_c_ represents the absorbance of the control without the sample solution, and A_s_ denotes the absorbance of the sample.

### Kinetic study of BChE inhibition

Kinetic studies were performed using the same protocol as the BChE inhibitory activity assay, and the substrate BTCI was used in a series of concentrations, 0.2, 0.4, 0.5, 0.6, and 0.7 mM. The test compound concentrations were set according to the inhibition IC_50_ of each compound. The enzymatic reaction was extended to 15 min before the detection of the absorbance. Lineweaver–Burk plots were generated to obtain the kinetic parameters, Michaelis constant (Km) and maximum velocity (Vmax), according to which inhibition modes were determined. The value of the inhibitor constant Ki was determined based on a secondary plot of K as a function of the inhibitor concentration [I] for competitive inhibitors (Copeland, 2000).

### Molecular docking and molecular dynamics studies

The docking calculations were performed with Autodock Vina software (Trott and Olson, 2010) to investigate the modes of combinations between BChE and the inhibitors. The 3D structures of the compounds were generated and then energetically minimized with an MM_2_ force field to a minimum root mean square (RMS) gradient of 0.005 using Chem3D Ultra 2017 (Version 17.0.0.206) (Wu et al., 2021). The crystal structures of human BChE and AChE were extracted from the Protein Data Bank (PDB code: 5k5e and 4ey4, respectively), and were further prepared by removing the water, ions, and original ligands. After the ligand and protein pdb files were prepared, AutoGrid was used to prepare the grid map using a grid box, with the grid size set to 26 × 26 × 26, and the grid box center was appointed to the Trp82 residue at coordinates x = −5.396, y = 6.442, and z = 16.584. All of the parameters were set to default values for the simulated annealing. The completed docking procedure produced nine top-ranked ligand-receptor conformations sorted based on the calculated free energy of binding. The best pose of each ligand with the highest affinity score (kcal/mol) was visualized using Discovery Studio Visualizer v21.1.0.20298 (Accelrys, San Diego, United States) for analyzing the interaction modes between the enzyme and inhibitors.

Following preliminary docking, the molecular dynamics of the complex between the enzyme and inhibitors was carried out by the PMEMD module in AMBER 20. For preparation, the docking modes of the inhibitors were applied with an AMBER ff99SB forcefield, and the general AMBER forcefield was used for the protein and ligand (Hornak et al., 2006). The simulation systems were solvated in a TIP3P water box in a 10 Å hexahedron, and the systems were neutralized by adding sodium ions. The simulation procedure was started with two steps of minimization, each set to 1,000 steps in order to reduce possible steric stresses. Then, the systems were heated to 300 K in a linear manner using a Langevin thermostat over 20 ps under NVT ensemble with weak restraints of 10 kcal/mol/Å^2^ on the protein backbone atoms. Subsequently, a step of equilibration under NPT ensemble was set at 1 atm and 300 K by the Langevin thermostat over 200 ps, followed by another one under NVT ensemble by a Berendson thermostat over 1 ns? Finally, the frames were extracted from 20 ns of the trajectory for the AMBER CPPTRAJ analysis (Roe and Cheatham., 2013). The binding free energy and its decomposition were calculated using the MMGBSA method (Ylilauri and Pentikainen., 2013) in AMBER 20.

## Results and discussion

### Fungal strain and active constituent screening for BChE inhibition

**FIGURE 1:**
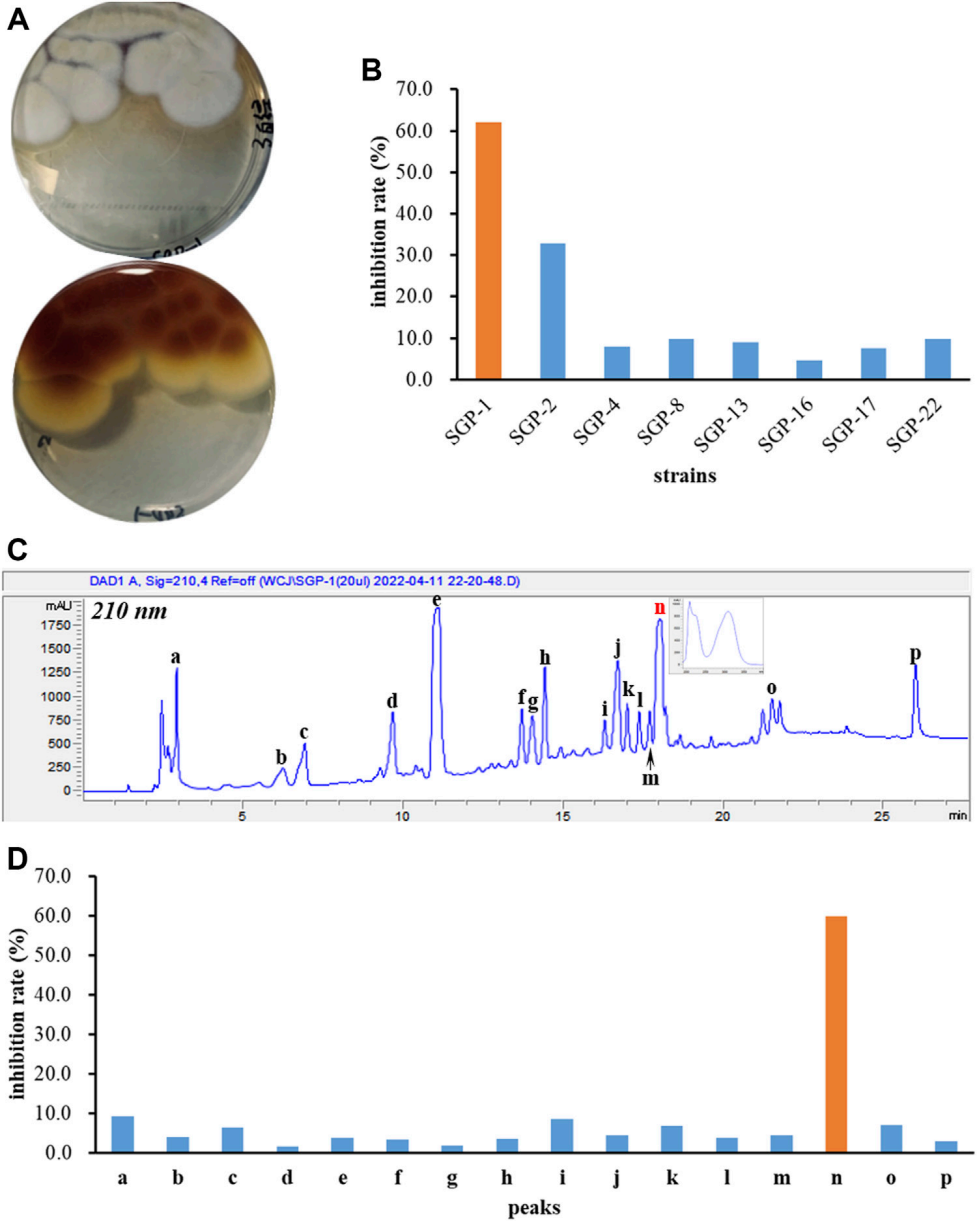
BChE inhibition screening of fungal strains and secondary metabolites. **(A)** Colonial morphology of *Aspergillus terreus* SGP-1; **(B)** BChE inhibitory activity of eight endophytic fungal strains; **(C)** HPLC preparation of the chemical constituents of SGP-1 EtOAc extract; **(D)** BChE inhibitory activity of SGP-1 chemical constituents.

Eight endophytic fungal strains, *Aspergillus terreus* SGP-1, *Fusarium solani* SGP-2, *Lecanicillium aphanocladii* SGP-4, *Penicillium janthinellum* SGP-8, *Fusarium nematophilum* SGP-13, *Mucor racemosus* SGP-16, *Acrocalymma vagum* SGP-17, and *Cladosporium colombiae* SGP-22 were isolated previously from the root bark of *Morus alba* L. and the EtOAc extracts of their fermentations were subjected to BChE inhibition screening assays. As a result, the *A. terreus* SGP-1 (Figure 1A) was found to be significantly inhibitory to BChE with an inhibition rate of 62.0% at 100 μg/ml (Figure 1B). In order to identify the representative bioactive constituents of the fermentation sample, a rapid micro-preparative HPLC separation was carried out. The eluates of the chromatographic peaks were collected, which were further diluted or concentrated to the same volume, so that the concentration of each fraction was equivalent to its concentration in the parent sample. The sample solutions with constant volumes were further tested for their BChE inhibitory activity. A total of 16 fractions, **a**–**p**, were collected (Figure 1C), and only fraction **n** was identified to be inhibitory to BChE (inhibitory rate% value of 59.8%, Figure 1D), suggesting that it was the main active constituent accounting for the BChE inhibitory activity of the SGP-1 EtOAc extract.

### Isolation and structure determination of butenolide derivatives

**FIGURE 2:**
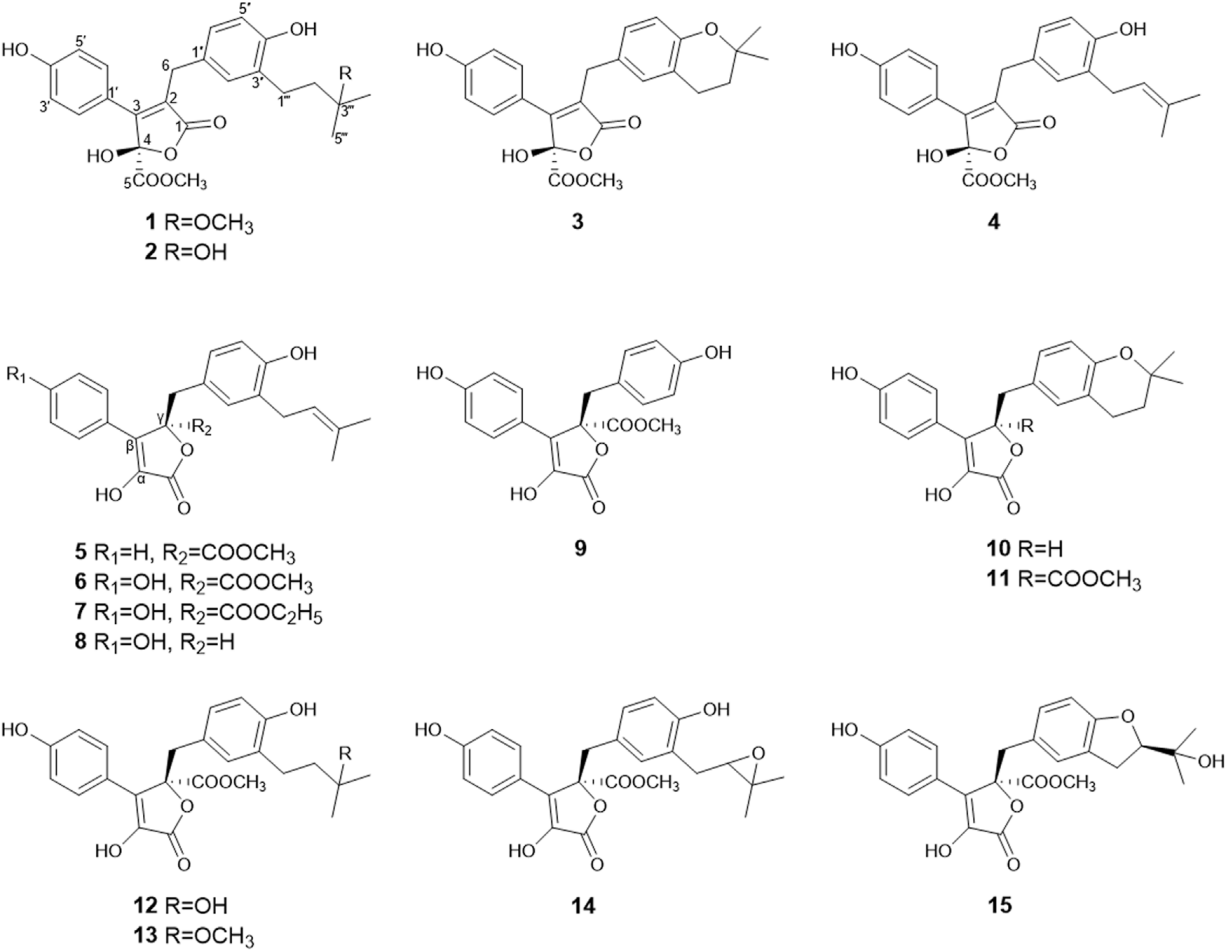
Chemical structures of compounds **1**–**15**.

In order to characterize the BChE inhibitory secondary metabolites, large scale fermentation and EtOAc extract preparation of strain SGP-1 and HPLC-guided separation were performed. The EtOAc extract of SGP-1 was separated using various types of CC and repeated preparative HPLC separations under HPLC-PDAD-UV monitoring with the characteristic UV spectra (the inset in Figure 1C) of the bioactive constituent as well as its analogs, resulted in the isolation of butenolide derivatives **1**–**15** (Figure 2). The known compounds were determined to be flavipesolide B (**3**) (Wang et al., 2016); butyrolactone VIII (**4**) (Ma et al., 2014); versicolactone B (**5**) (Qi et al., 2018); butyrolactone I (**6**) (Rao et al., 2000); butyrolactone VII (**7**) (Haritakun et al., 2010); 3-hydroxy-5-[[4-hydroxy-3-(3-methyl-2-buten-1-yl) phenyl]methyl]-4-(4-hydroxyphenyl)-2(5H)-furanone (**8**) (Guo et al., 2016); butyrolactone II (**9**) (Rao et al., 2000); 5-[(3,4-dihydro-2,2-dimethyl-2H-1-benzopyran-6-yl)methyl]-3-hydroxy-4-(4-hydroxyphenyl)-2(5H)-furanone (**10**) (Kiriyama et al., 1977); aspernolide A (**11**), B (**12**), and C (**13**) (Parvatkar et al., 2009); butyrolactone III (**14**) (Nitta et al., 1983); and butytolactone IV (**15**) (Rao et al., 2000), by comparison of the MS and NMR data with that of the literature. Among them, butyrolactone I (**6**) was one of the most abundant constituents in the EtOAc extract of SGP-1, and denoted as the fraction **n** using HPLC analysis.

**FIGURE 3:**
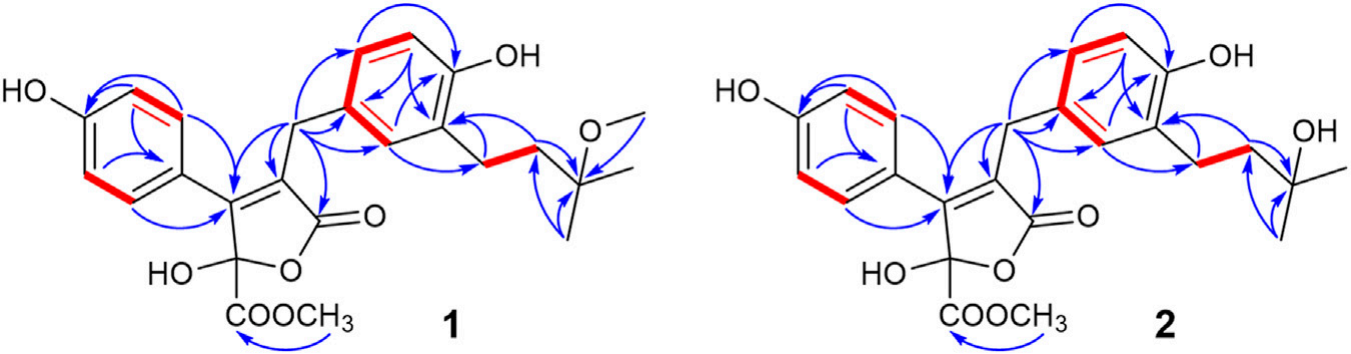
Key COSY and HMBC correlations of compounds **1**–**2**.

Compound **1** was obtained as a pale-yellow solid. The molecular formula was determined to be C_25_H_28_O_8_ on the basis of a high-resolution electrospray ionization mass spectroscopy (HRESIMS) peak at m/z 479.1678 [M + Na]^+^ (calcd. 479.1682), indicating 12 degrees of unsaturation. The UV absorptions at 209, 226, and 310 nm were similar to those of **4**, indicating the same butenolide skeleton. The presence of a *p*-substituted phenyl moiety was suggested by the ^1^H NMR signals of δ_H_ 7.48 (d, J = 8.49 Hz, 2H) and 6.78 (d, J = 8.9 Hz, 2H), as well as a 1,3,4-trisubstituted benzene ring indicated by three aromatic hydrogen signals at δ_H_ 6.90 (d, J = 2.3 Hz, 1H), 6.85 (dd, J = 8.2, 2.3 Hz, 1H), and 6.67 (d, J = 8.2 Hz, 1H), combined with the correlated spectroscopy (COSY) correlations of H-2′, 6′ (δ_H_ 7.48)/H-3′, 5′ (δ_H_ 6.78), and H-2″ (δ_H_ 6.90)/H-5″ (δ_H_ 6.67)/H-6″ (δ_H_ 6.85) (Figure 3). According to the distortionless enhancement by polarization transfer (DEPT) and heteronuclear single-quantum correlation (HSQC) spectra, the ^13^C NMR spectrum showed 25 carbon signals, including four methyls, three methylenes, seven sp^2^-methines, and 11 quaternary carbons. The heteronuclear multiple bond correlation (HMBC) spectra exhibited long-range correlations (Figure 3) from H-2'' (δ_H_ 6.90) and H-6″ (δ_H_ 6.85) to C-6 (δ_C_ 30.1), from H-2″ to C-6'' (δ_C_ 127.4) and oxygenated C-4″ (δ_C_ 154.9), and from H-5″ (δ 6.67) to C-1'' (δ_C_ 129.1)/C-3'' (δ_C_ 130.5), indicating the 1,3,4-trisubstituted benzene ring existed as a 3,4-disubstituted benzyl group. There were two methylene signals of H-1‴ (δ_H_ 2.57–2.50, m, 2H), and H-2‴ (δ_H_ 1.72–1.65, m, 2H) exhibiting strong COSY correlations with each other, and a gem-dimethyl signal of δ_H_ 1.19 (s, 6H). Thus, a 3‴-methoxy isopentyl was deduced, combining with HMBC signals from H-1‴ to C-3‴ (δ_C_ 76.4), from 3‴-OCH_3_ (δ_H_ 3.21) to C-3‴, and from H-4‴/H-5‴ (δ_H_ 1.19) to C-2‴ (δ_C_ 40.4). Furthermore, the HMBC correlations from H-1‴ to C-2″–C-4″ and from H-2‴ to C-3″ linked the 3‴-methoxy isopentyl to the benzyl group at C-3″. The HMBC correlations from H_2_-6 (δ_H_ 3.77) to C-1 (δ_C_ 174.2), C-2 (δ_C_ 127.3), and C-3 (δ_C_ 157.0) connected the benzyl group to the γ-butenolide nucleus through C-2. The *p*-substituted phenyl was linked to C-3 of the γ-butenolide nucleus according to the key HMBC correlations from H-2′ and H-6′ to C-3 (δ_C_ 157.0). The downfield chemical shift of C-4 (δ_C_ 103.1) suggested a ketal or hemiketal carbon connecting with a methoxycarbonyl on the basis of the HMBC correlation of a methoxy proton (δ_H_ 3.74) to a carbonyl carbon C-5 (δ_C_ 169.3). As yet, there are two hydrogen and two oxygen atoms remaining, implying that C-4 should be a hemiketal carbon, and the *p*-substituent group of the phenyl is a hydroxy. Thus, the chemical construction of compound **1** was elucidated as methyl-2-hydroxy-4-(4-hydroxy-3-(3-methoxy-3-methylbutyl)benzyl)-3-(4-hydroxyphenyl)-5-oxo-2,5-dihydrofuran-2-carboxylate, and named as asperteretal J.

Compound **2** was also obtained as a pale-yellow solid, and its molecular formula was deduced as C_24_H_26_O_8_ from the HRESIMS peak at *m/z* 465.1526 [M + Na]^+^, indicating one CH_2_ unit less than **1**. The ^1^H and ^13^C NMR spectral data (Table 1) were similar to those of **1**, except for the absence of one oxygenated methyl signal. This suggested that compound **2** was the demethyl analog of **1**, while the remaining oxygenated methyl was linked to C-5 according to the key HMBC between methoxy protons (δ_H_ 3.75) and the carbonyl carbon C-5 (δ_C_ 169.3). Accordingly, compound **2** was elucidated as methyl-2-hydroxy-4-(4-hydroxy-3-(3-hydroxy-3-methylbutyl)benzyl)-3-(4-hydroxyphenyl)-5-oxo-2,5-dihydrofuran-2-carboxylate, and named as asperteretal K.

Compounds **1** and **2**, as well as flavipesolide B (Wang et al., 2016) and asperteretal B (Guo et al., 2016), could be regarded as the derivatives of butyrolactone VIII (**4**), sharing the γ-butenolide nucleus with the same substitution pattern and possessing the single chiral center C-4. The absolute structure of C-4 in previously reported natural analogs was determined to be an *S*-configuration by the electron-capture dissociation method along with negative specific optical rotation ([α]) values (Guo et al., 2016; Wang et al., 2016). In this study, compounds **1** and **2** also showed levorotation at 589.3 nm with [α]values of −18.4 and −17.5, respectively, suggesting they had the *S*-configuration of C-4.

Phenyl- and benzyl-disubstituted γ-butenolides are typical secondary metabolites produced by fungi, especially *Aspergillus* sp., while they are rarely found in plants and animals (Ibrahima et al., 2017). According to the substituted positions on the γ-butenolide nucleus of the phenyl and benzyl groups, their structures can be divided into three types, α,β-, β,γ-, and α,γ-disubstituted γ-butenolides, all biosynthesized originally from *p*-hydroxyphenylpyruvic acid (HPPA), or phenylpyruvic acid (PPA) through different dimerization modes. The β,γ- and α,γ-disubstituted types are common, natural γ-butenolides with butyrolactone I (**6**) and aspulvinone H as typical structures, respectively. As for rare α,β-disubstituted types, the representative compound was butyrolactone VIII (**4**). This series of natural product theoretically possesses rich structural diversity resulting from prenylation at different positions, followed by hydration, oxidation, or cyclization of the olefinic bond, as well as decarboxylation or alkylation of the hydroxyl group. There are abundant and structurally diverse natural products belonging to 3,4- and 2,4-disubstituted γ-butenolides that have been reported from *Aspergillus* sp. (Ibrahima et al., 2017), while only a few examples for α,β-disubstituted types were reported (Ma et al., 2014; Guo et al., 2016; Wang et al., 2016). This study provided two new chemical entities for this rare natural product.

### ChE inhibitory activities

To identify potent ChE inhibitors, all compounds were subjected to BChE and AChE inhibitory activity screening. As a result, compounds **4**–**8** were found to significantly inhibit BChE at 100 µM with IR% values in the range of 60.9%–76.0% (72.4% for galantamine at 100 µM), and the IR% for the other compounds were approximately 30% or less, indicating an IC_50_ greater than 100 µM (Table 2), including aspulvinone H which was prepared previously from *A. terreus* ASM-1 (Wu et al., 2021). The IC_50_ values for compounds **4**–**8** were further determined to range from 18.4 to 45.8 μM as moderate BChE inhibitors. Butyrolactone I (**6**, IC_50_ = 35.5 μM) contributed to the inhibitory activity of SGP-1 crude EtOAc extract, while butyrolactone VII (**7**) was the most potent compound among them with an IC_50_ value of 18.4 μM. However, none of the compounds exhibited an inhibitory effect against AChE at 100 μM, suggesting that compounds **4**–**8** were selective BChE inhibitors.

**TABLE 2:** Inhibitory activities against ChEs of compounds **1**–**15**.

Cpd	AChE IR, %^a^	BChE IR, %^a^	BChE IC_50_ (µM)^b^	K_i_ (µM)^c^
**1**	11.2 ± 3.6	15.6 ± 2.3	>100	NT
**2**	7.7 ± 1.0	3.2 ± 3.6	>100	NT
**3**	19.6 ± 4.2	22.5 ± 3.1	>100	NT
**4**	11.9 ± 1.0	67.3 ± 2.8	45.8 ± 6.9	23.6
**5**	10.4 ± 1.9	64.4 ± 3.2	44.3 ± 3.7	38.2
**6**	−6.7 ± 3.6	68.0 ± 4.1	35.5 ± 1.3	19.3
**7**	11.7 ± 1.4	76.0 ± 3.5	18.4 ± 2.5	12.3
**8**	14.2 ± 0.5	60.9 ± 0.6	45.1 ± 7.2	35.7
**9**	9.6 ± 0.5	19.9 ± 6.0	>100	NT
**10**	23.2 ± 1.1	33.3 ± 7.5	>100	NT
**11**	−0.1 ± 0.7	25.4 ± 3.7	>100	NT
**12**	2.2 ± 1.5	13.4 ± 1.3	>100	NT
**13**	3.9 ± 0.9	22.6 ± 5.3	>100	NT
**14**	−4.2 ± 0.3	-12.3 ± 4.6	>100	NT
**15**	13.3 ± 5.5	33.5 ± 2.3	>100	NT
Aspulvinone H	9.6 ± 3.4	6.5 ± 2.6	>100	NT
Galantamine^d^	NT	72.4 ± 1.2	35.3 ± 5.6	NT

^a^ Compounds were tested at the concentration of 100 μM, the tests for each sample were performed in triplicate.

^b^ Sample concentration that led to 50% enzyme activity loss.

^c^ Ki is the inhibition constant.

^d^ Galantamine is used as the positive control.

NT, is not tested.

**FIGURE 4:**
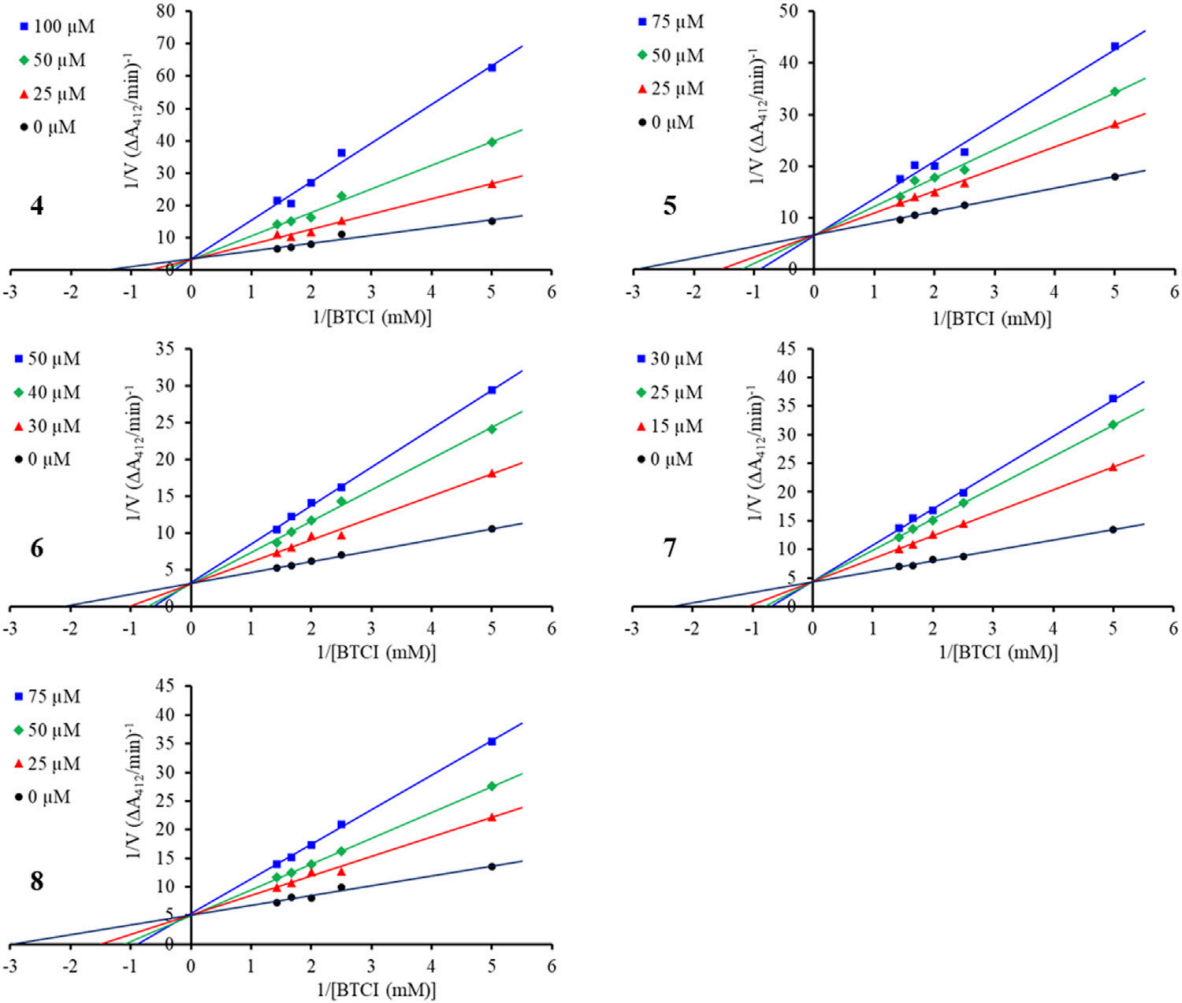
Lineweaver—Burk plot for BChE inhibition by compounds **4**–**8**.

Enzyme kinetic studies were carried out for compounds **4**–**8** to determine their inhibition modes. As shown in Figure 4, in the Lineweaver-Burk double-reciprocal plots, the plots of 1/V versus 1/[S] give a group of straight lines with different slopes that intersect at the y-axis for all inhibitors with constant V_max_ and enhanced K values as the dose of the inhibitors increased, suggesting that they are competitive inhibitors (Copeland, 2000). The secondary plot of K as a function of inhibitor concentration [I] exhibited good linearity (*R* ^2^ > 0.95) for each of the active compounds (Supplementary Figure S1), and the inhibition constants (Ki) under the experimental conditions were calculated (Table 2).

Comparing the structures and activities, the structure-activity relationship (SAR) may provide clues as follows. Whether the α,β- or β,γ-disubstituted types, the linear prenyl (without oxidation, hydration, or cyclization) was vital (**4** vs*.* **1**–**3**; **5**–**8** vs*.* **9**–**15**); *p*-hydroxyl on the phenyl moiety (**5** vs*.* **6**), as well as the methoxycarbonyl group linked to the γ-butenolide nucleus (**6** vs*.* **8**) contributed little; while extending the alkyl chain of the alkoxycarbonyl or enhancing its hydrophobicity may be beneficial (**6** vs*.* **7**); and the phenyl and benzyl substituted at two adjacent carbons of the γ-butenolide nucleus was essential for BChE inhibitory activity (**4** and **6** vs*.* aspulvinone H). These SAR clues may provide some guidance for structural modifications to obtain more potent selective BChE inhibitors with butyrolactone I (**6**) as a foundation, which has a high yield in *A. terreus* fermentation. Moreover, the phenyls and butenolide cycle in the structure could also be replaced by various kind Nitrogen-heterocycles for novel designed molecules to be totally synthesized. N-heterocycles have unique biological activity, low toxicity and high internal absorption, and are often used as structural units of pharmaceuticals and biologically interesting molecules, and play an important role in the synthesis of pharmaceuticals due to their unique structural characteristics (Kim and Movassaghi, 2009; Arslan et al., 2019; Staśkiewicz et al., 2021). Compounds containing aromatic N-heterocycles, such as triazole (Arslan et al., 2019; Özil et al., 2019), pyridine (Arslan et al., 2020), pyrimidine (Cavdar et al., 2019) were reported with potent BChE inhibitory activity, suggesting the nitrogen of N-heterocycles may provide powerful interactions with the residues of catalytic pocket of the enzyme.

### Molecular docking

**FIGURE 5:**
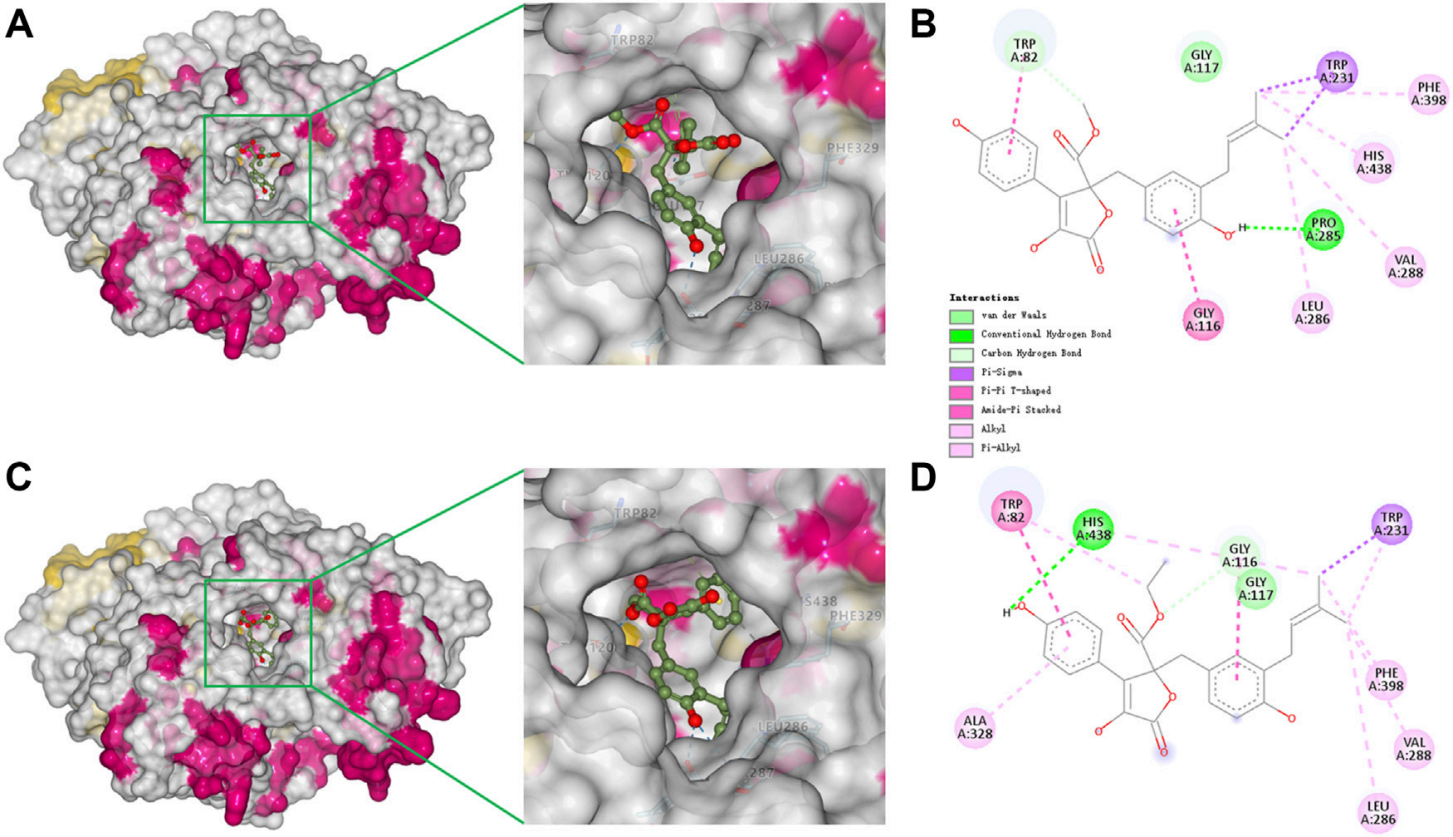
Docking binding model of inhibitors with BChE (5k5e). Predicted dock conformation of 5k5e to inhibitors **6 (A)** and **7 (C)**; 2D interaction diagrams between 5k5e and inhibitors **6 (B)** and **7 (D)**.

To better understand the capacity of butanolide derivatives to bind with BChE, the binding modes of compounds **6** and **7** were investigated using Autodock Vina. Both compounds could insert into the binding groove of BChE successfully, forming diverse interactions with the residues of the enzyme (Figures 5A,C). There are some common interactions of BChE with both compounds **6** and **7**: the benzyl ring forms a π-amide stacked interaction with Gly116; the phenyl ring binds with Trp82 by π-π T-shaped interactions; the prenyl forms a π-σ interaction with Trp231; and there are π-alkyl or alkyl interactions with Leu286, Val288, and Phe398. The structural difference between **6** and **7** leads to diverse interactions with BChE (Figures 5B, D). As for compound **6**, the benzyl *p*-hydroxy forms a conventional hydrogen bond with Pro285, and prenyl forms an additional π-alkyl interaction with His438. As for compound **7**, the hydrogen bond is formed between the phenyl *p*-hydroxy and His438, and there is an additional π-alkyl interaction between the phenyl ring and Ala328. Moreover, the key residue Trp82 interacts with the methyl of the methoxycarbonyl group of **6** through van der Waals forces, but with the ethyl group of **7** by a stronger π-alkyl effect. This diversity in binding modes may lead to the differences in the inhibition potencies against BChE. The catalytic pocket of BChE is a ∼20 Å deep gorge, with subdomains including a midgorge aromatic recognition site, a peripheral anionic site, an acyl-binding pocket (Trp231, Leu286, Val288), a catalytic triad (Ser198, His438, Glu325), and a choline binding pocket (Trp82) (Nicolet et al., 2003; Brus et al., 2014). Molecular docking results displayed that compounds **6** and **7** could properly occupy the catalytic active site of BChE contributing to their competitive inhibitory activities.

Because compounds **4**–**8** selectively inhibit BChE, molecular docking of **7** with AChE (PDB code: 4ey4) was also conducted to discover the structure-based reason for target selectivity. Human AChE and BChE share 65% homological amino acid sequences, displaying a similar overall structure. Their acyl-binding pockets displayed remarkable difference (Nicolet et al., 2003; Cheung et al., 2012). The acyl-binding pocket residues Phe288 and Phe290 in AChE are replaced by Leu286 and Val288 in BChE, so as to accommodate bulkier ligands and substrates. Additionally, it was reported that larger residues of AChE (Phe297 and Tyr124) protrude into the pocket, and result in the formation of a narrower pocket in AChE, while smaller residues of BChE (Gln119 and Val286) make the pocket broader (Davis et al., 1997). Consistently, the docked complex of AChE and **7** indicated that compound **7** with a rigid structure barely inserted into the bottom of the gorge (Supplementary Figure S2), which might contribute to the inefficiency of the inhibition to AChE.

### Molecular dynamics simulation

**FIGURE 6:**
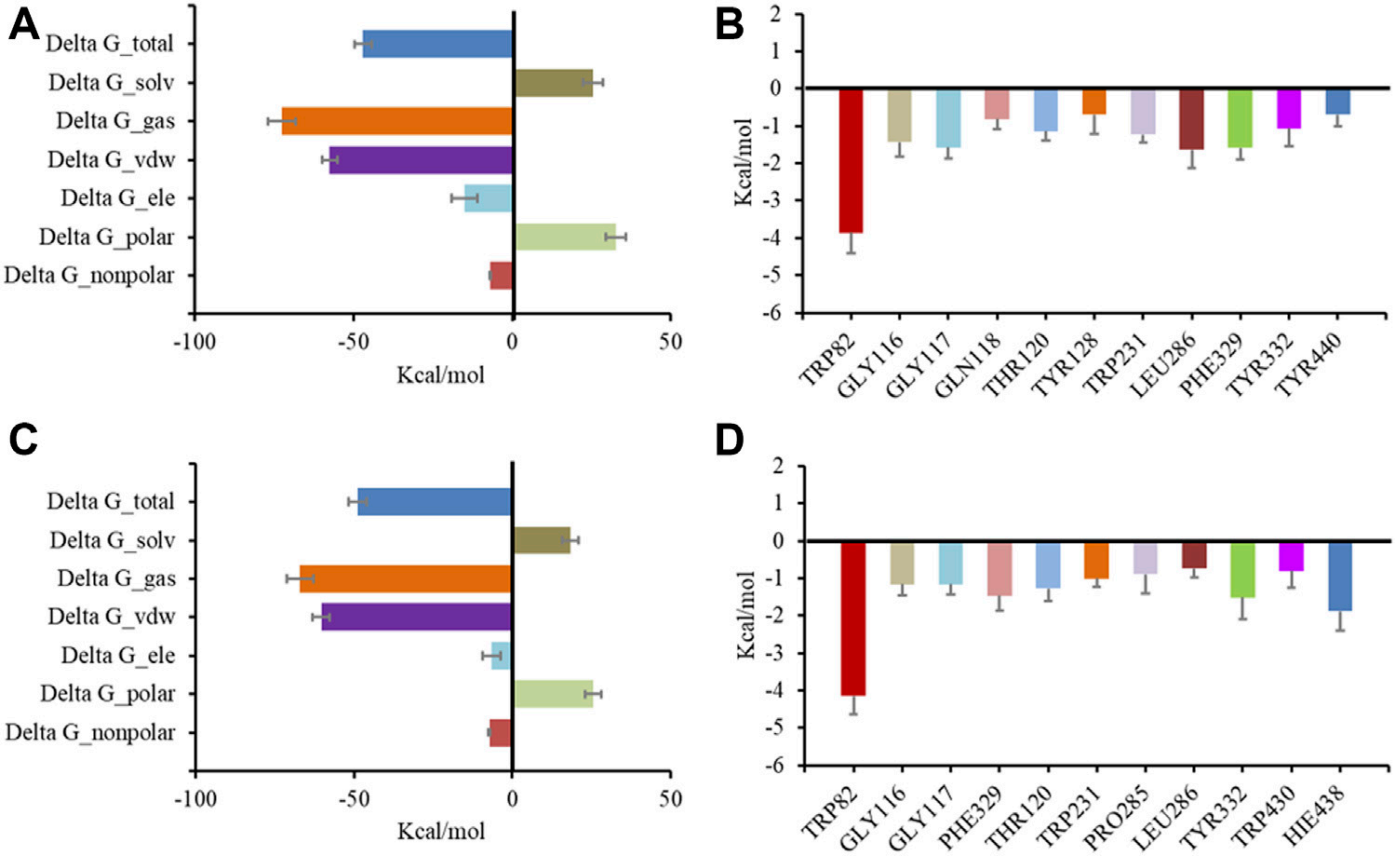
Molecular dynamic simulation results for BChE with inhibitors. Total binding free energy and its component for **6 (A)** and **7 (C)** with 5k5e; residue contribution to receptor-ligand complexes for 5k5e with **6 (B)** and **7 (D)**.

Following preliminary docking, a molecular dynamics simulation was conducted in AMBER to evaluate the stability of the complex of BChE and the inhibitors. Both **6** and **7** combined with BChE steadily in 20 ns. Calculations by the MMGBSA method gave the total binding free energy as −41.28 kcal/mol and −48.85 kcal/mol for the complex of BChE-**6** and BChE-**7**, respectively (Figures 6A, C). The structures of compounds **4**–**8** are of high rigidity, and the poor flexibility of the molecules may produce a low binding free energy with BChE. Among the binding free energies, van der Waals energy (Δ G_vdw, −51.71 kcal/mol for **6**, and −60.43 kcal/mol for **7**) was the most important component. Previous studies found that π-π interactions of the inhibitor with Trp82, Trp231, and Phe329, and a hydrogen bond between the molecule and His438 were significant residues for binding to BChE (Nicolet et al., 2003; Nachon et al., 2013; Knez et al., 2015). To identify the key residues in BChE for binding **6** and **7**, the contributions of hot residues in the binding pocket of BChE were analyzed. The residues with interaction energies lower than −1 kcal/mol are considered to be essential for ligand recognition and complexing. As a result, Trp82 (−3.86 kcal/mol), Leu286 (−1.64 kcal/mol), Phe329 (−1.59 kcal/mol), Gly117 (−1.57 kcal/mol), Gly116 (−1.43 kcal/mol), Thr120 (−1.14 kcal/mol), and Tyr332 (−1.07 kcal/mol) are regarded as key residues for compound **6** binding with BChE. The key residues for **7** are Trp82 (−4.14 kcal/mol), His438 (−1.89 kcal/mol), Trp231 (−1.23 kcal/mol) Tyr332 (−1.52 kcal/mol), Phe329 (−1.46 kcal/mol), Thr120 (−1.27 kcal/mol), Gly116 (−1.16 kcal/mol), Gly117 (−1.15 kcal/mol), and Trp231 (−1.01 kcal/mol) (Figures 6B, D). Consistent with the docking results, the Trp82 residue demonstrated more contribution to the binding through interaction with the ethyl of **7**.

Butyrolactone I (**6**) can be produced by *A. terreus* derived from various environmental samples or endophytic hosts (Ibrahim et al., 2017). As one of the most abundant products of *A. terreus*, butyrolactone I bioactivity has been researched through a series of *in vitro* and *in vivo* studies. Butyrolactone-I was identified as an efficient α-glucosidase inhibitor (IC_50_ = 1.52 µM), improved type 2 diabetes in db/db mice and showed potent TNF-α-lowering properties by modulating gut microbiota. As a cyclin CDC-2 kinase as well as CDK-2 kinase inhibitor (Atkinson et al., 1993), butyrolactone I inhibited tumor cell proliferation (Nishio et al., 1996), and induced cancer cell apoptosis (Wada et al., 1998). Additionally, it has antibacterial effects and anti-inflammatory activities that inhibited the secretion of NO and TNF-α in macrophages and microglia (Li et al., 2014). Moreover, it has been shown to alleviate inflammatory responses in lipopolysaccharide-stimulated IPEC-J2 and DSS-induced murine colitis through the TLR4/NF-κB and MAPK signal pathway, and might potentially be used as therapy to prevent intestinal bowel disease (Chen et al., 2021).

As for neuroprotective effects, butyrolactone I prevented apoptosis of central and peripheral neurons (Maas et al., 1998), induced neuronal differentiation of PC12 cells (Dobashi et al., 2000), alleviated the toxic effect of Aβ on human neuroblastoma cells (SH-SY5Y) by inhibiting cyclin-dependent kinase 5 (CDK5) (Wei et al., 2002), significantly downregulated tau protein hyperphosphorylation in rat hippocampal primary neurons induced by the pro-inflammatory factor interleukin 6 (IL-6) (Quintanilla et al., 2004), and had good anti-neuroinflammatory effects (50 µM) on lipopolysaccharide-induced mouse microglia (BV-2 cells) and mouse monocyte macrophages (RAW 264.7 cells) (Zhang et al., 2018). Most recently, butyrolactone I was reported to ameliorate AlCl_3_-induced cognitive deficits in zebrafish in a dose-dependent manner, reversed the elevated levels of central and peripheral proinflammatory cytokines, and increased AChE activity in the brains of the zebrafish induced by AlCl_3_ at the dosage of 50 mg/kg (Nie et al., 2022). The present study exhibited the BChE inhibitory activity of butyrolactone I for the first time, which may provide a new explanation for its neuroprotective activities. The previous studies together with our findings suggested that butyrolactone I may be applied as multi-target therapy for preventing central nervous system deficits including AD through anti-inflammatory, CDK inhibitory, and neurotrophic effects (Unzeta et al., 2016), as well as the cholinomimetic effect.

## Conclusion

In summary, we screened fungal strains and their constituents for BChE inhibitors, and an *Aspergillus terreus* SGP-1 and its main product butyrolactone I (**6**) was found to have potent and selective inhibitory activities toward BChE based on HPLC analyses combined with bioassays. Further chemical investigation led to the isolation of a total of fifteen butenolide derivatives from the SGP-1 culture, including two new compounds. Their structures, including the absolute configurations, were elucidated by various spectroscopic methods and optical rotation comparison. All compounds were evaluated for AChE and BChE inhibition with galantamine as a positive control. Among them, compounds **4**–**8** selectively inhibited BChE rather than AChE in a competitive manner, with Ki values of 12.3–38.2 µM. The SAR analysis suggested some chemical modifications or biomimetic synthesis based on butyrolactone I (**6**) may underlay the more potent BChE inhibitors. The molecular docking and dynamic simulation studies recognized that the inhibitors could successfully insert into the binding groove of BChE, forming diverse interactions with the residues of the target, particularly Trp82. The present study provides a new perspective for understanding the neuroprotective effects of butyrolactone I.

## Data Availability

The datasets presented in this study can be found in online repositories. The names of the repository/repositories and accession number(s) can be found in the article/Supplementary Material.
