# Supplementary material for: Butenolide derivatives from Aspergillus terreus selectively inhibit butyrylcholinesterase
Source: Front Chem. 2022 Dec 21;10:1063284. doi: 10.3389/fchem.2022.1063284 (PMC9811682; doi:10.3389/fchem.2022.1063284)
Supplement: Supplementary file 1 [file DataSheet1.PDF]

Supplementary Material

Butenolide derivatives from *Aspergillus terreus* selectively inhibit butyrylcholinesterase

Xiang Cui[1,2], Shanshan Deng[2], Guoyin Li[2], Yunxia Zhang[2], Lining Wang[1], Changjing Wu[2*] and Yanru Deng[1*]

[1]College of Traditional Chinese Medicine, Tianjin University of Traditional Chinese Medicine, Tianjin, China

[2]College of Life Sciences and Agronomy, Zhoukou Normal University, Zhoukou, China

[*]Correspondence:
Changjing Wu
wucj2009@163.com

Yanru Deng
dengyanru@tjutcm.edu.cn

List of Supplementary Materials

Figure S1. Secondary plot of K_m^{app} as a function of inhibitor concentration for BChE inhibition 1
Figure S2. Comparison of docking models of butyrolactone VII (**7**) with AChE (a) and BChE (b) . 2
Figure S3. HRESIMS data of compound **1** 3
Figure S4. ^{1}H-NMR spectrum of compound **1** in CD_3OD 4
Figure S5. ^{13}C-NMR and DEPT-135 spectra of compound **1** in CD_3OD 4
Figure S6. HSQC spectrum of compound **1** in CD_3OD 5
Figure S7. ^{1}H-^{1}H COSY spectrum of compound **1** in CD_3OD 5
Figure S8. HMBC spectrum of compound **1** in CD_3OD 6
Figure S9. HRESIMS data of compound **2** 7
Figure S10. ^{1}H-NMR spectrum of compound **2** in CD_3OD 8
Figure S11. ^{13}C-NMR spectra of compound **2** in CD_3OD 8
Figure S12. HSQC spectrum of compound **2** in CD_3OD 9
Figure S13. ^{1}H-^{1}H COSY spectrum of compound **2** in CD_3OD 9
Figure S14. HMBC spectrum of compound **2** in CD_3OD 10

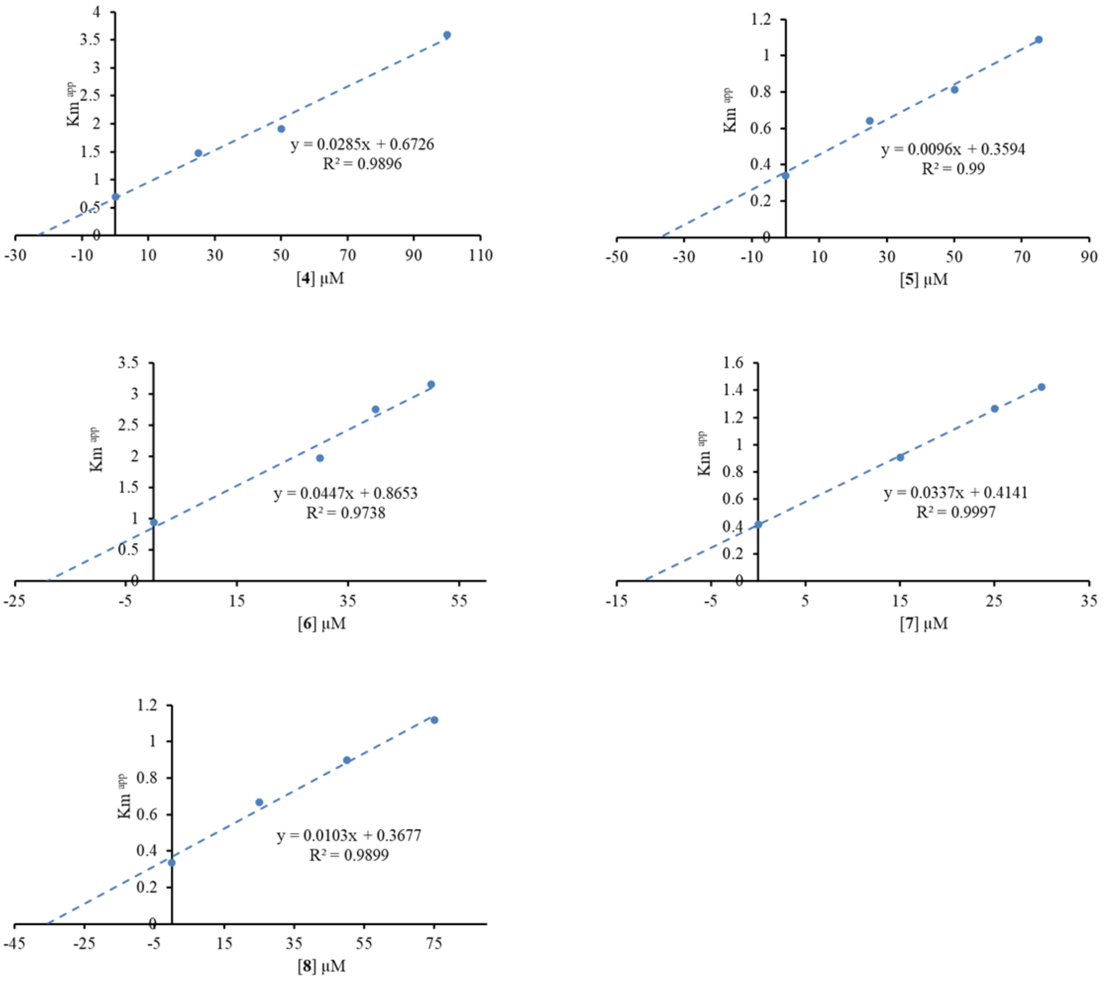

Figure S1. Secondary plot of K_m^{app} as a function of inhibitor concentration for BChE inhibition

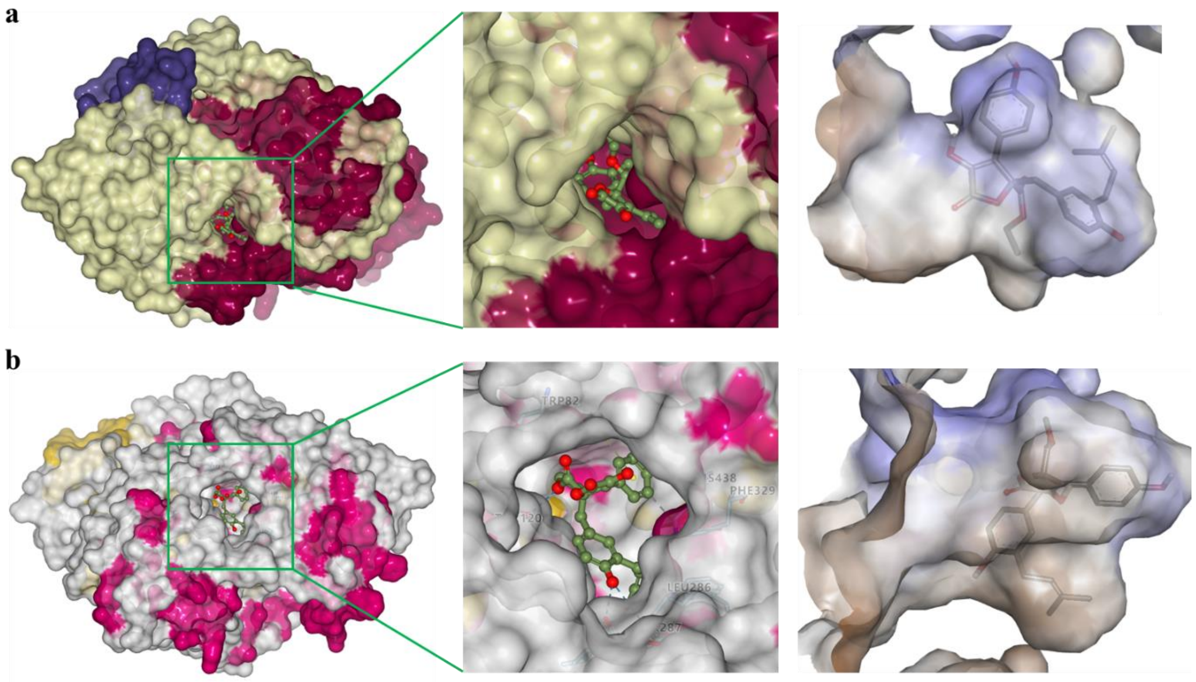

Figure S2. Comparison of docking models of butyrolactone VII (**7**) with AChE (a) and BChE (b).

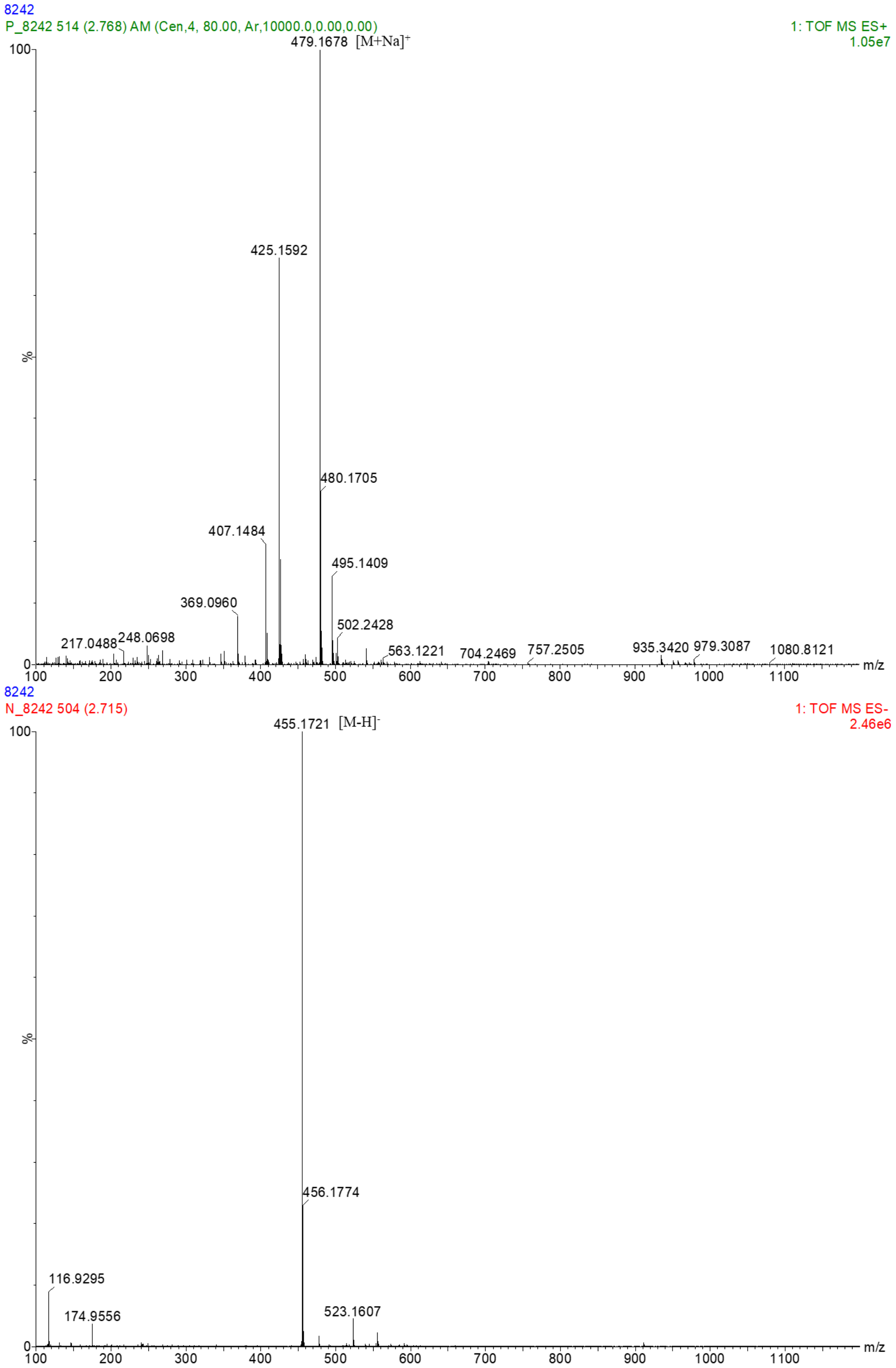

Figure S3. HRESIMS data of compound **1**

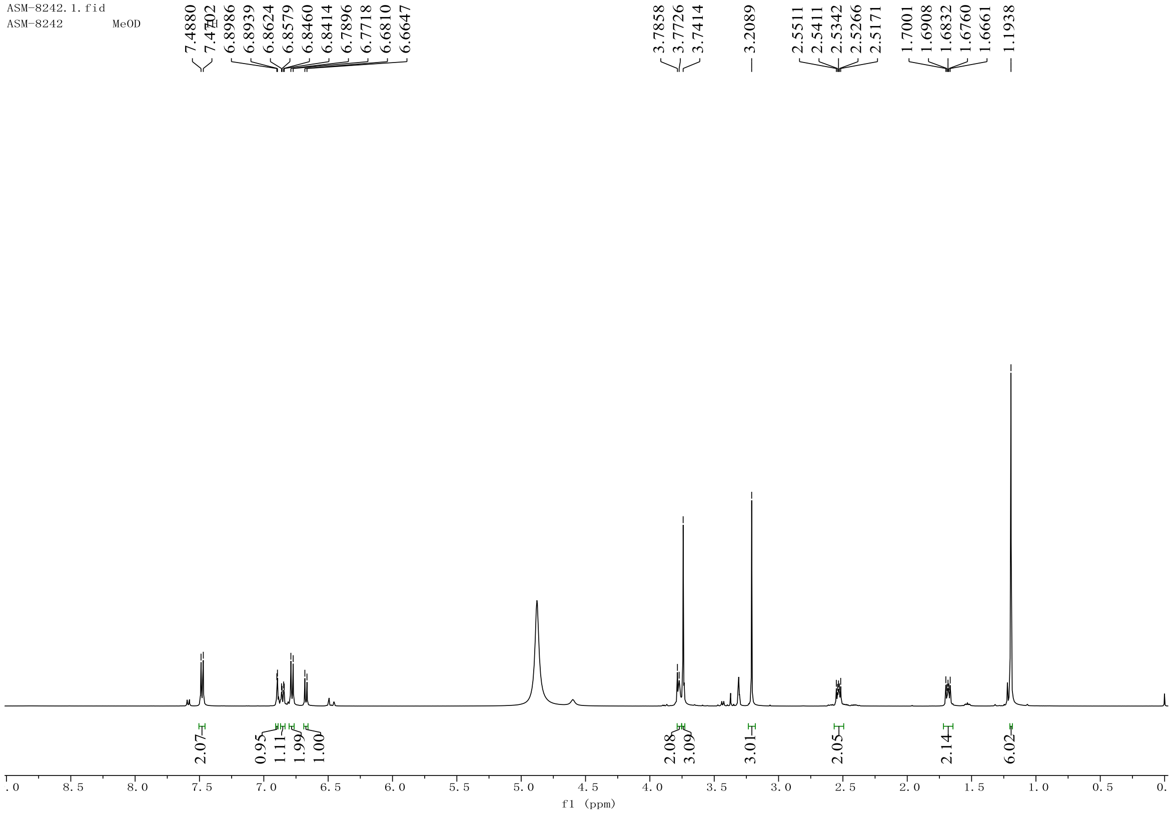

Figure S4. ^{1}H-NMR spectrum of compound **1** in CD_3OD

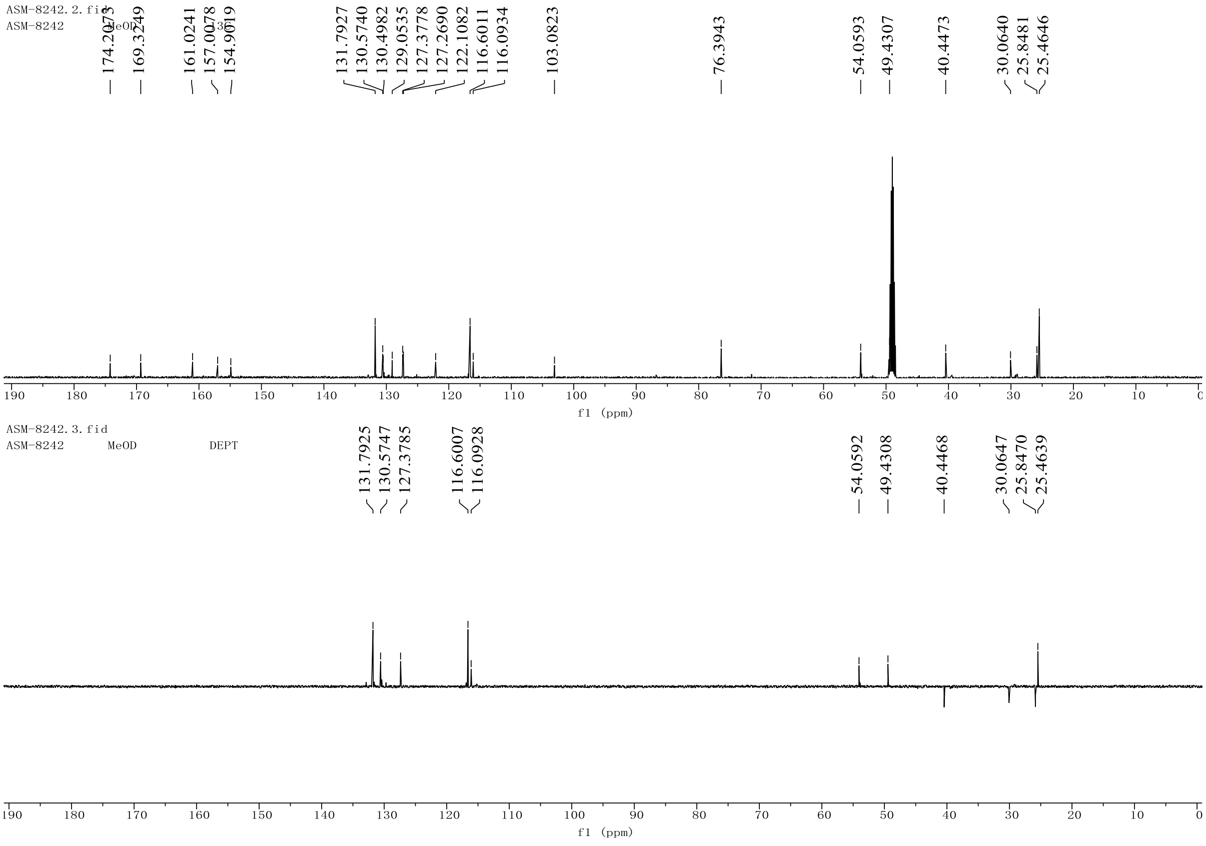

Figure S5. ^{13}C-NMR and DEPT-135 spectra of compound **1** in CD_3OD

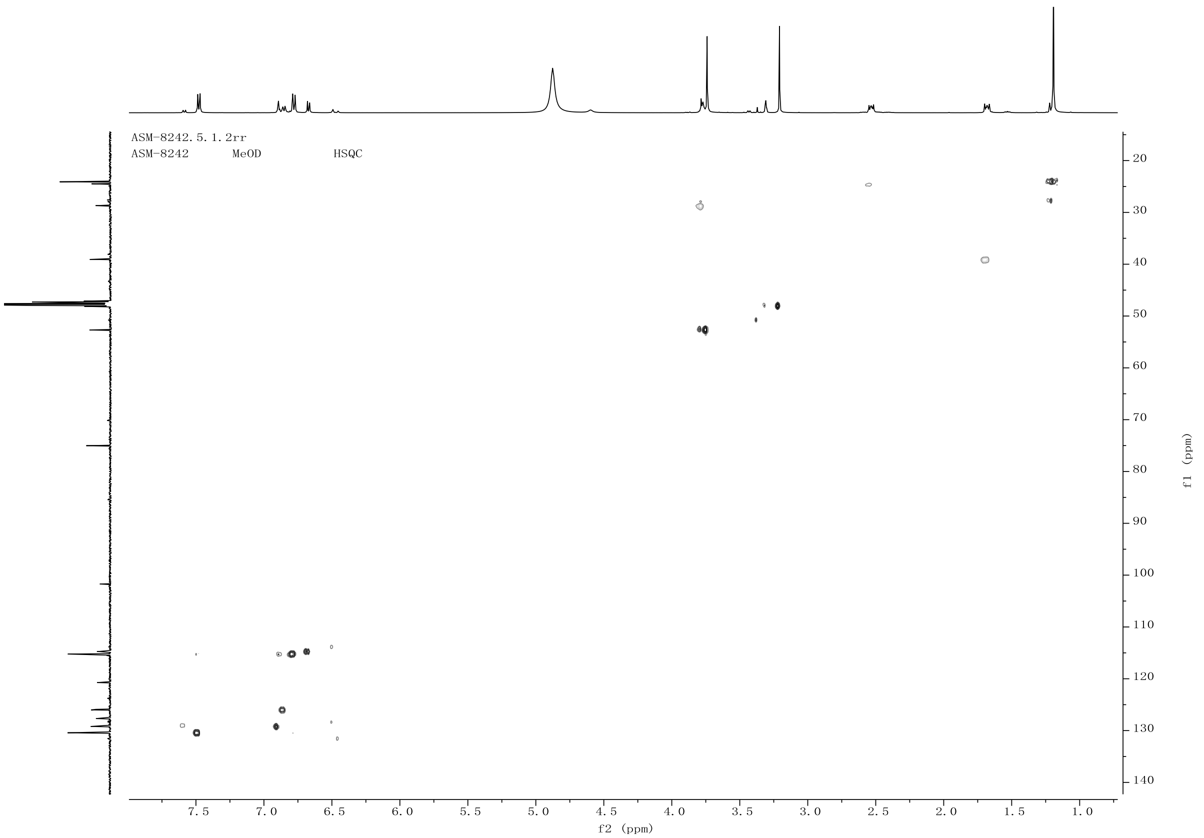

Figure S6. HSQC spectrum of compound **1** in CD_3OD

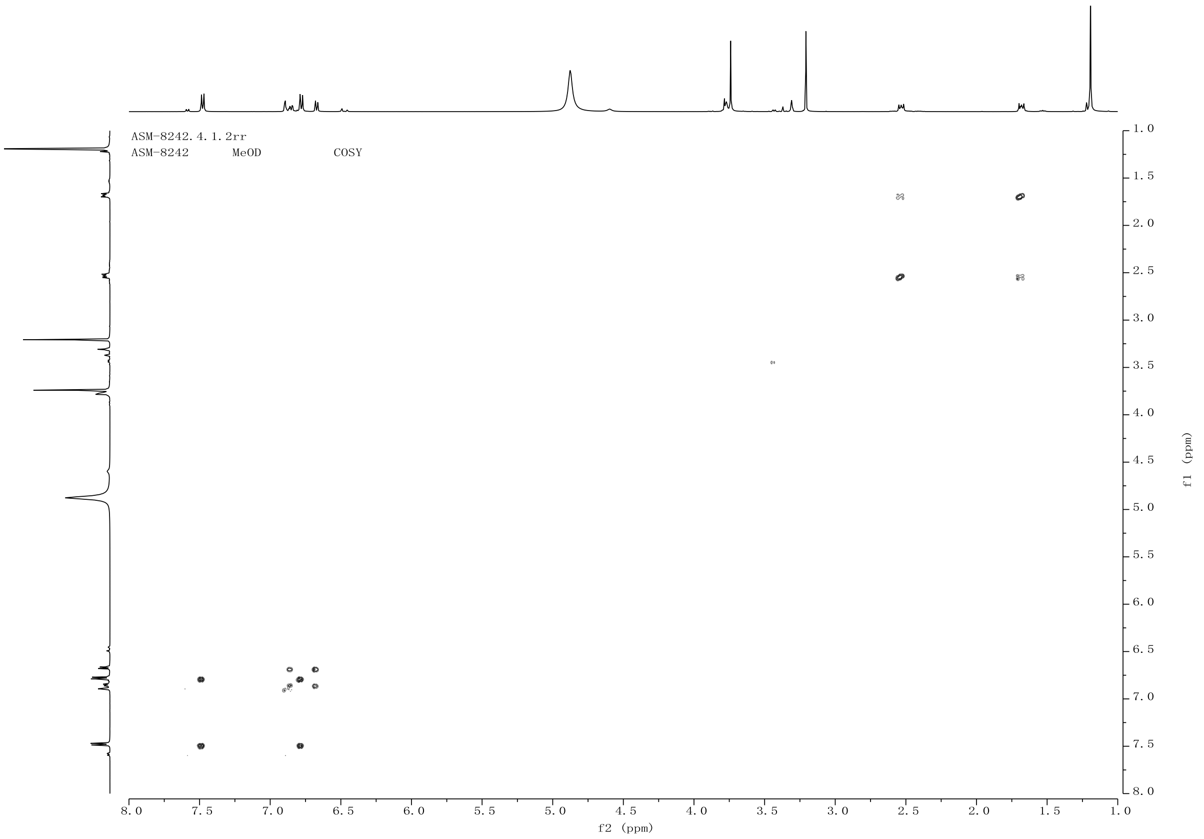

Figure S7. ^1H-^1H COSY spectrum of compound **1** in CD_3OD

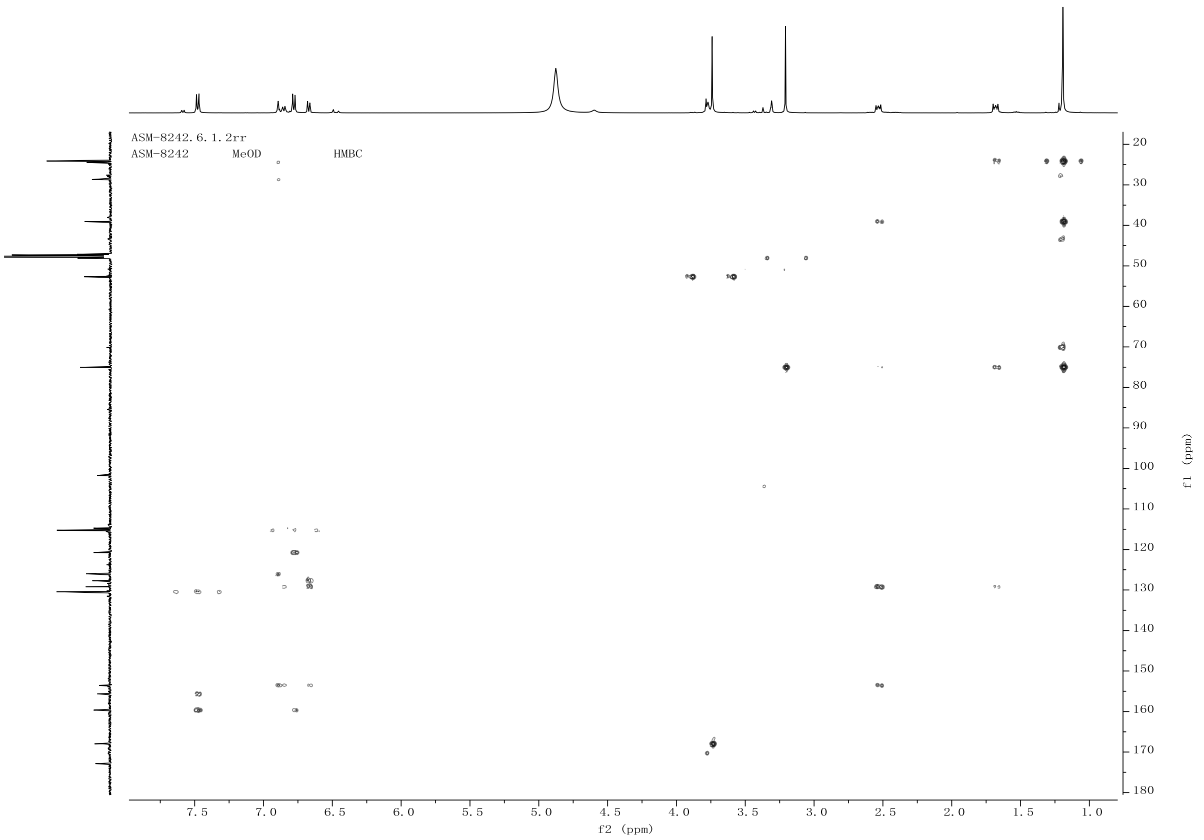

Figure S8. HMBC spectrum of compound **1** in CD_3OD

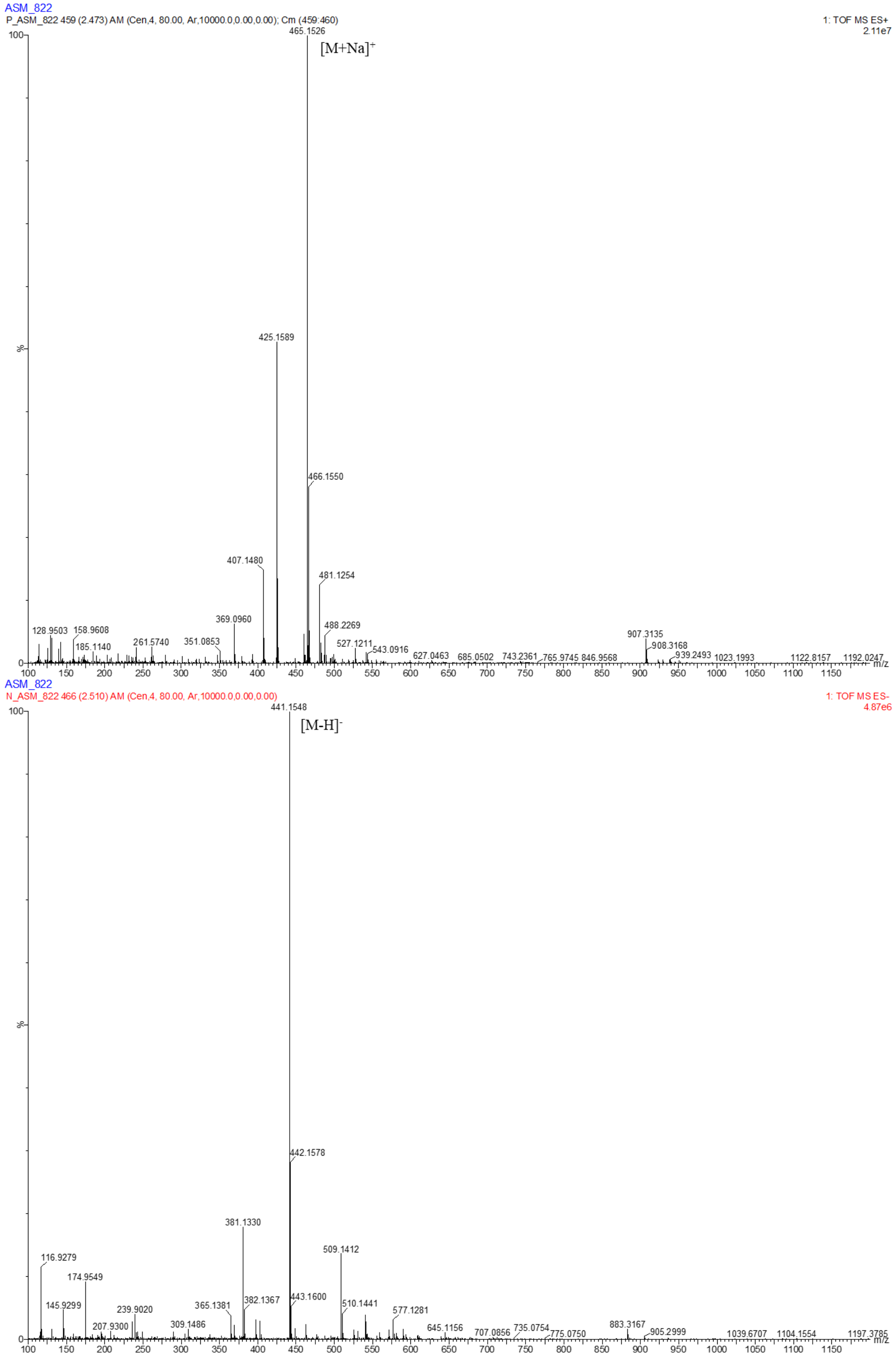

Figure S9. HR-ESI-MS data of compound **2**

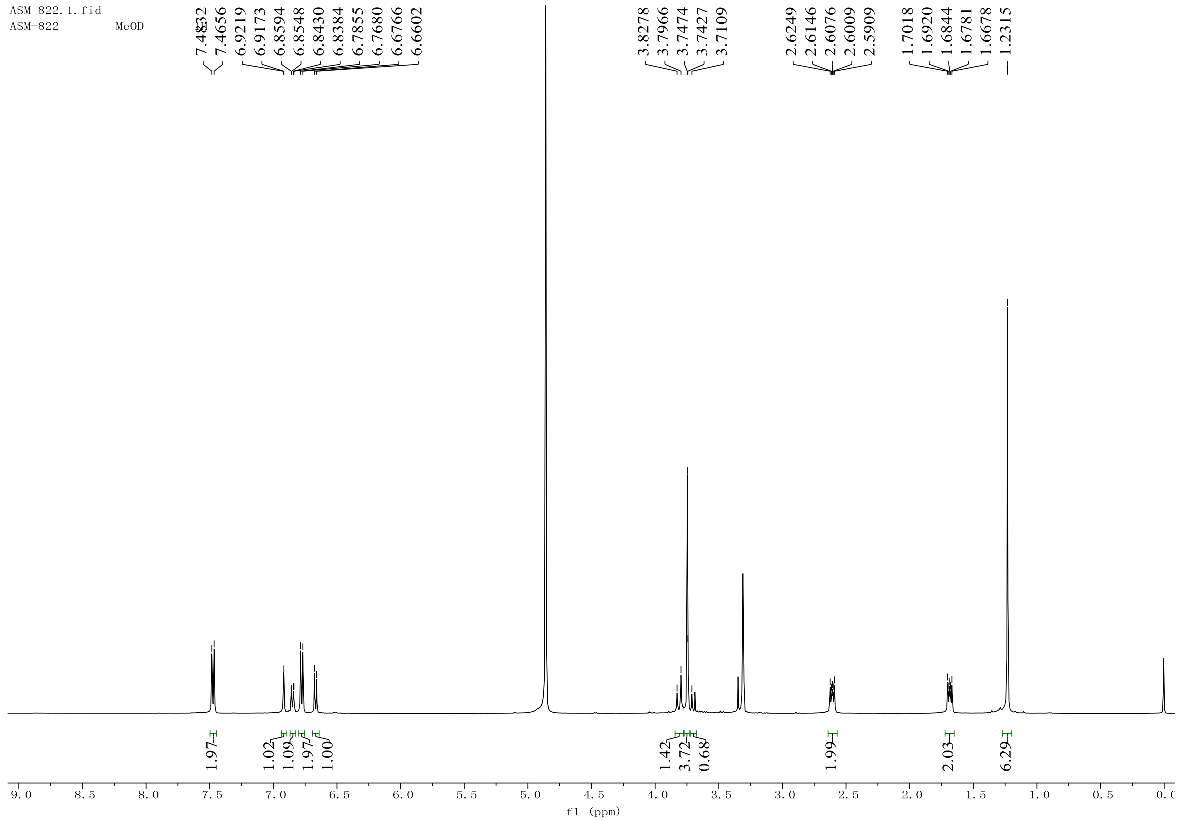

Figure S10. ^{1}H-NMR spectrum of compound **2** in CD_3OD

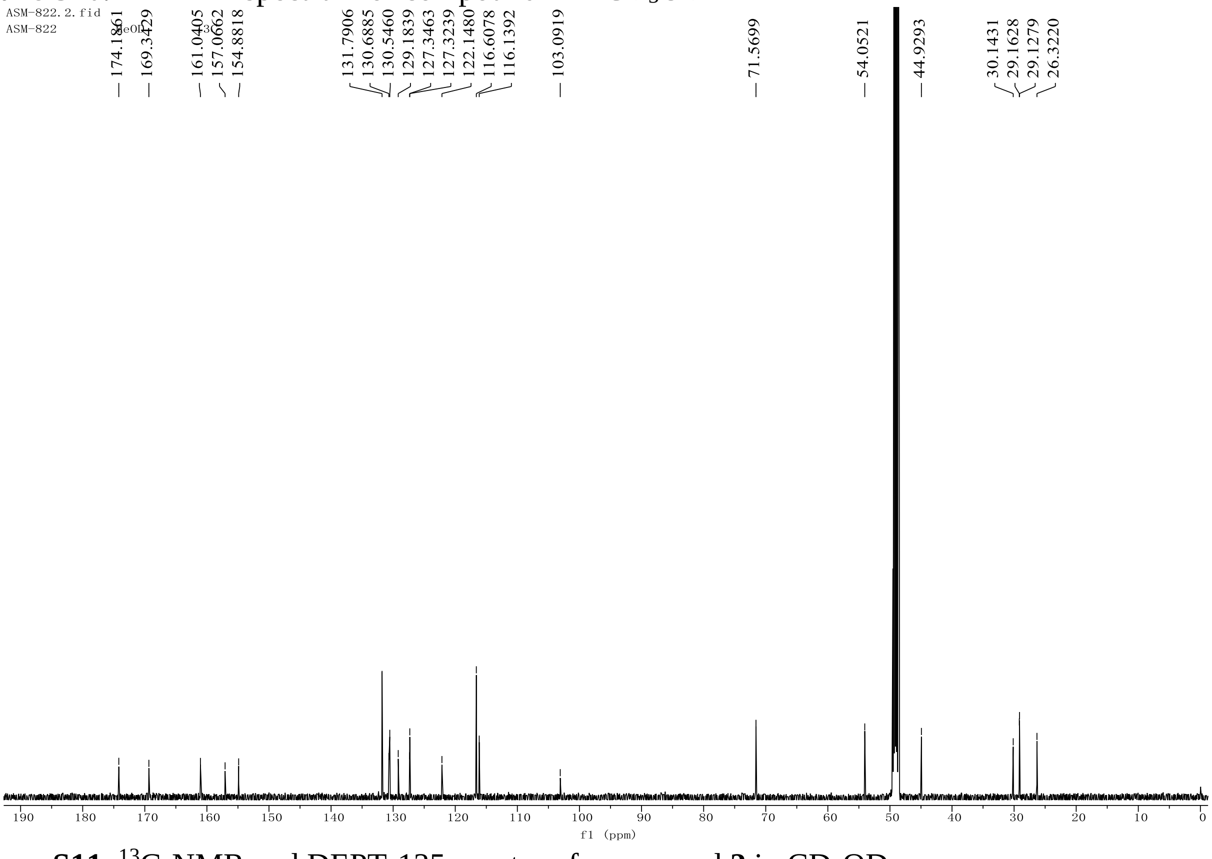

Figure S11. ^{13}C-NMR and DEPT-135 spectra of compound **2** in CD_3OD

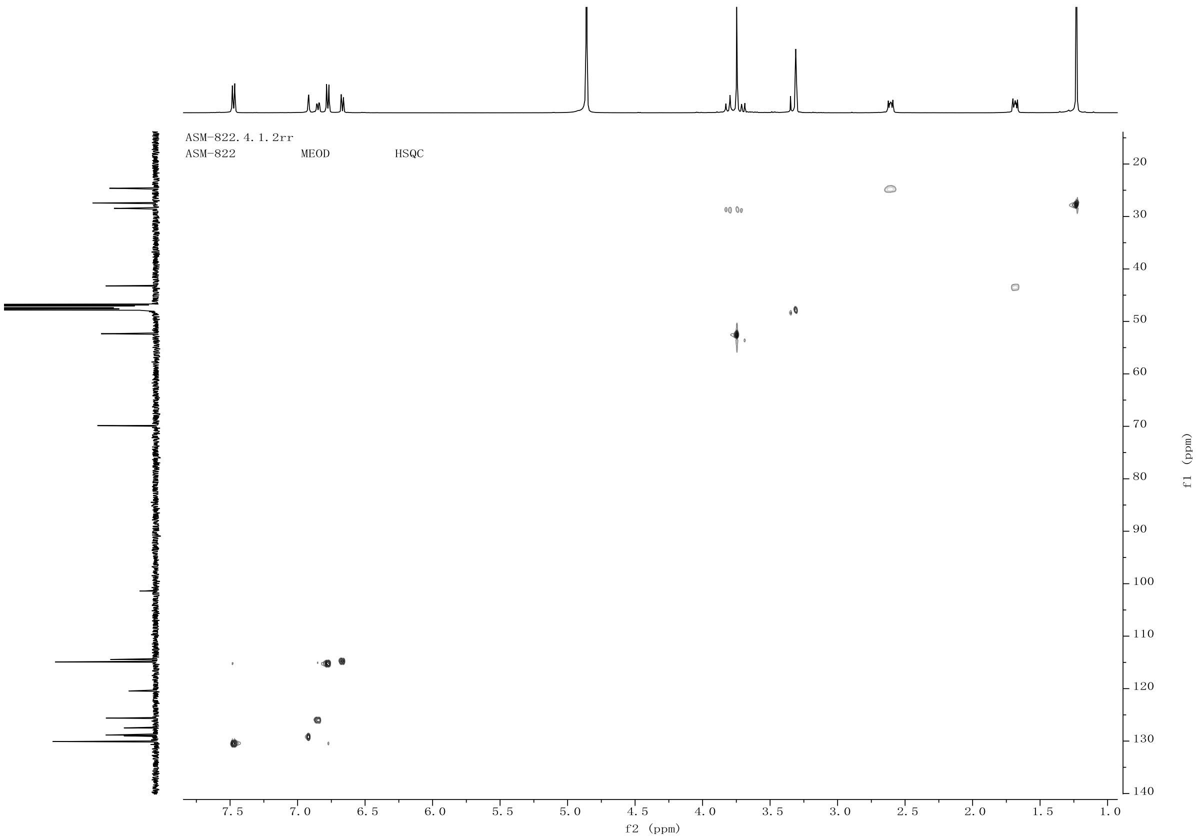

Figure S12. HSQC spectrum of compound **2** in CD_3OD

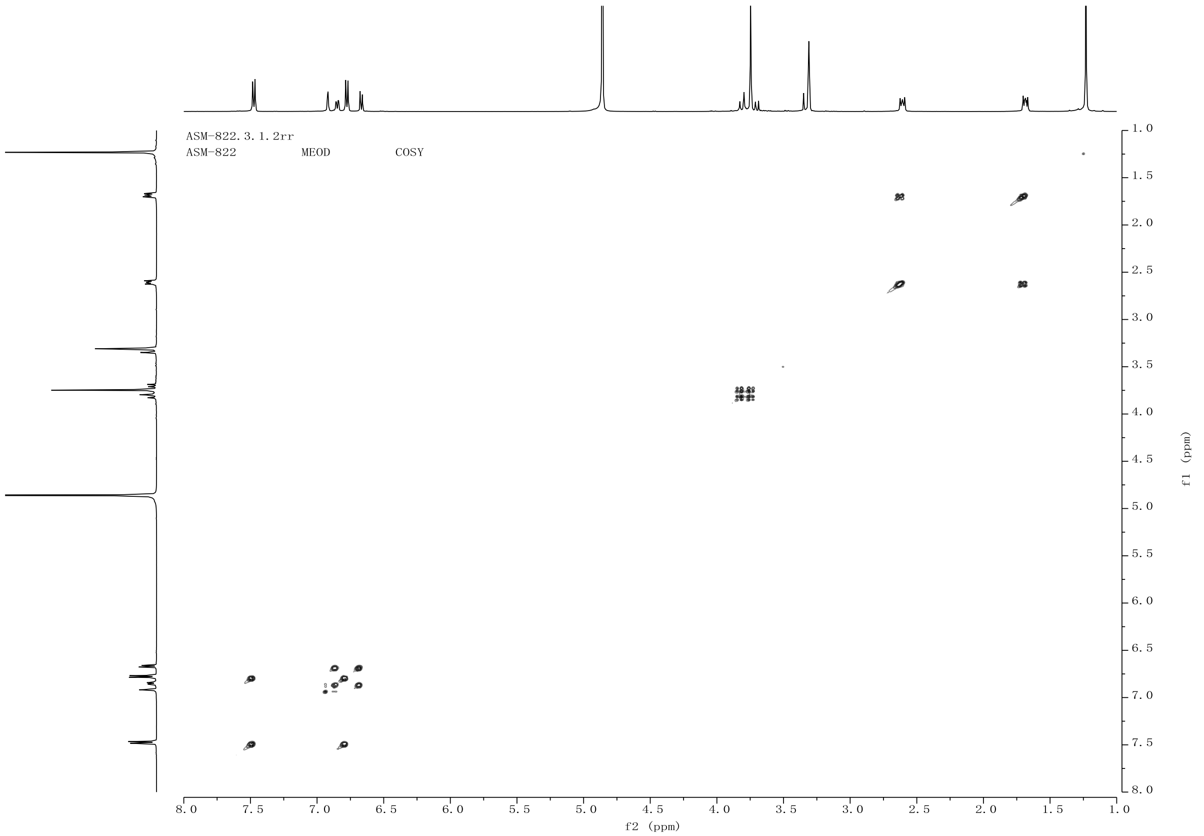

Figure S13. ^1H-^1H COSY spectrum of compound **2** in CD_3OD

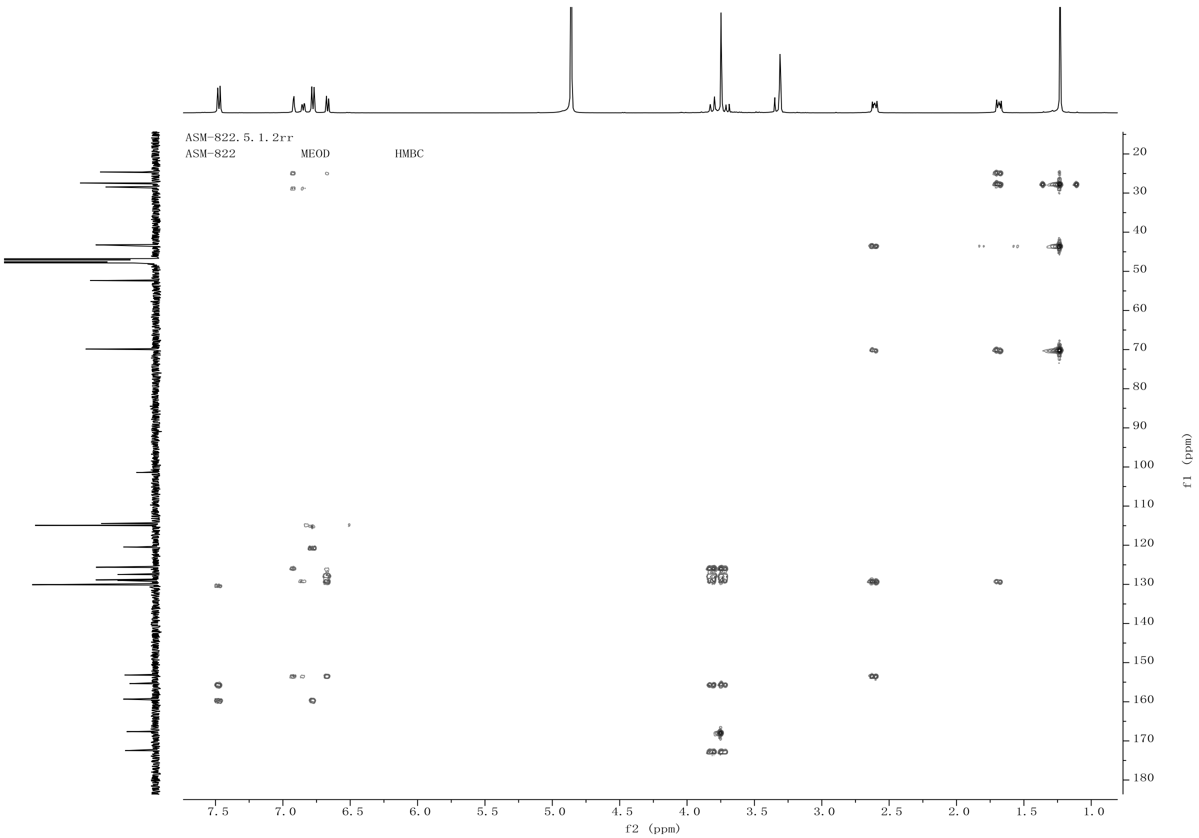

Figure S14. HMBC spectrum of compound **2** in CD_3OD
